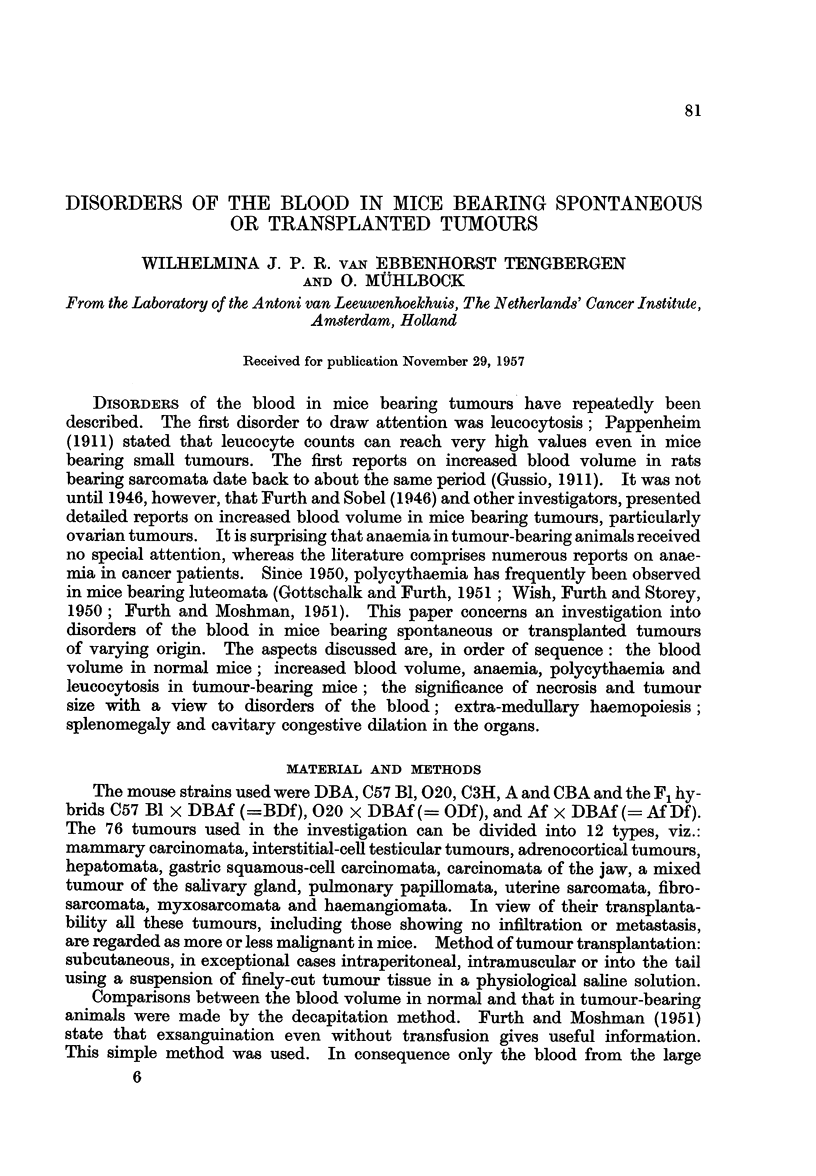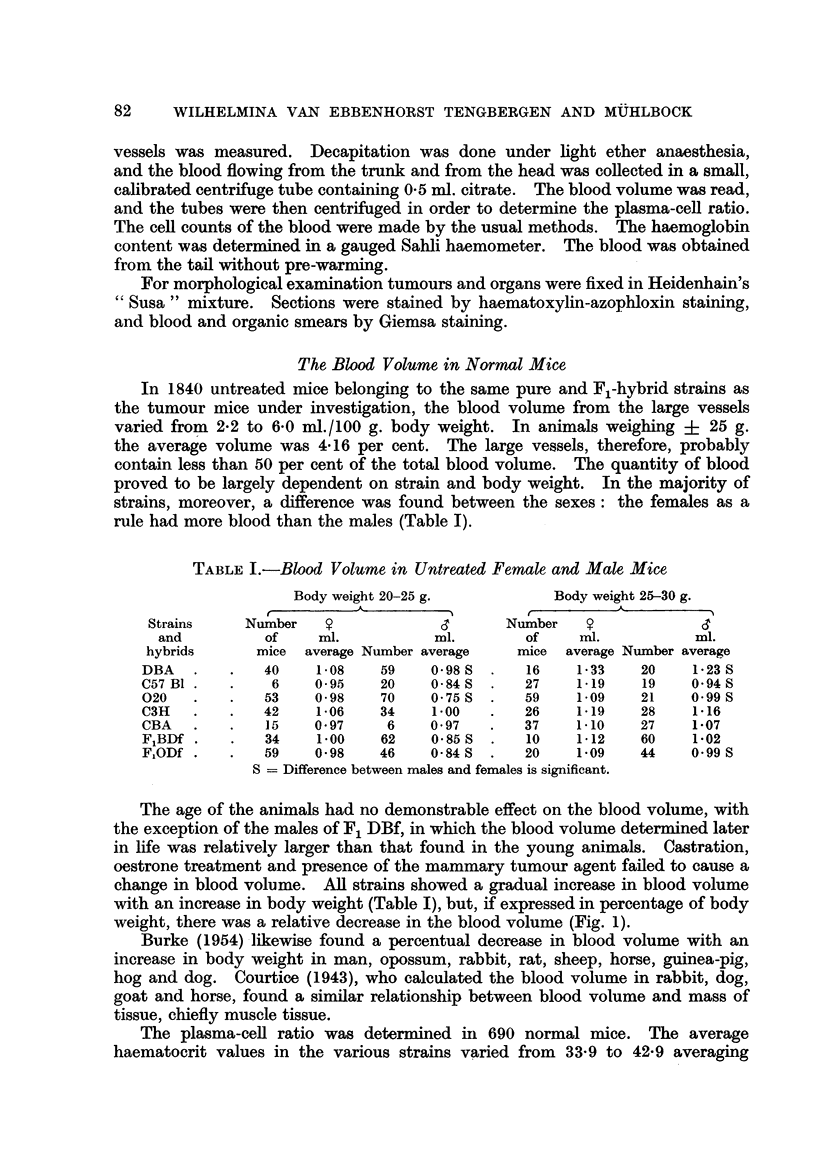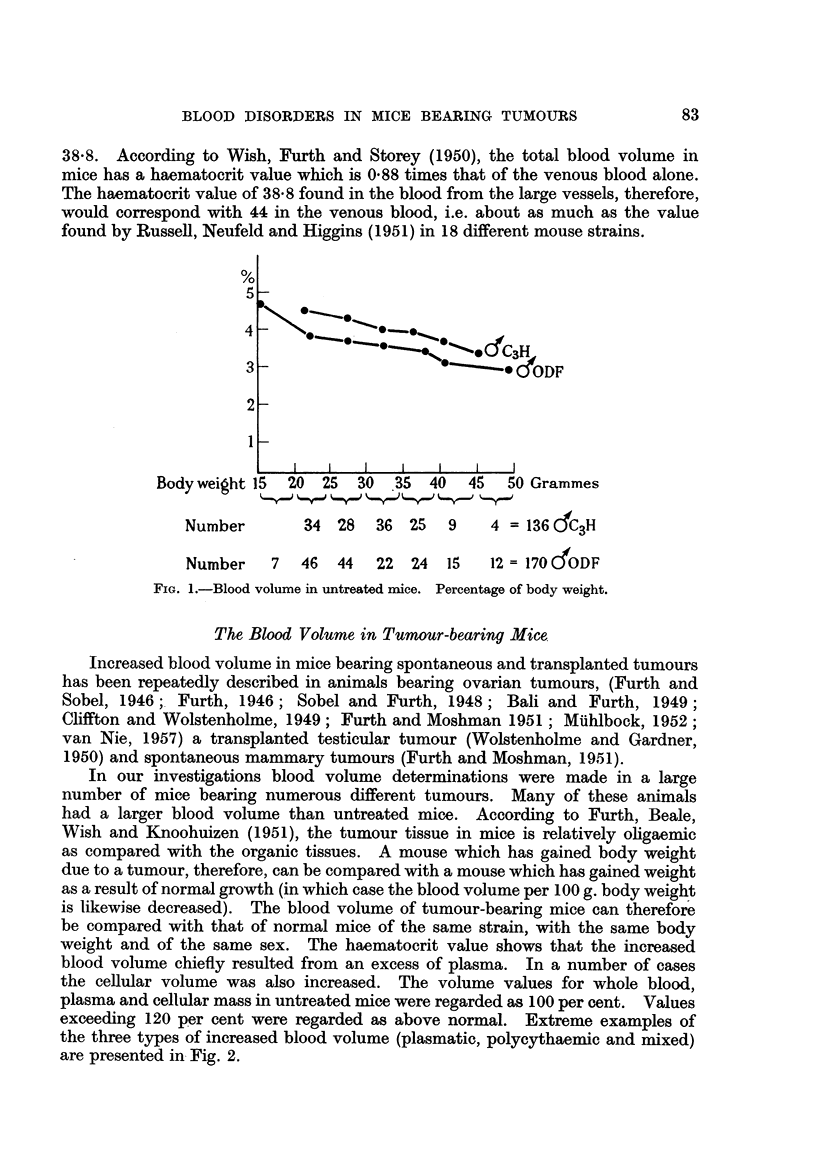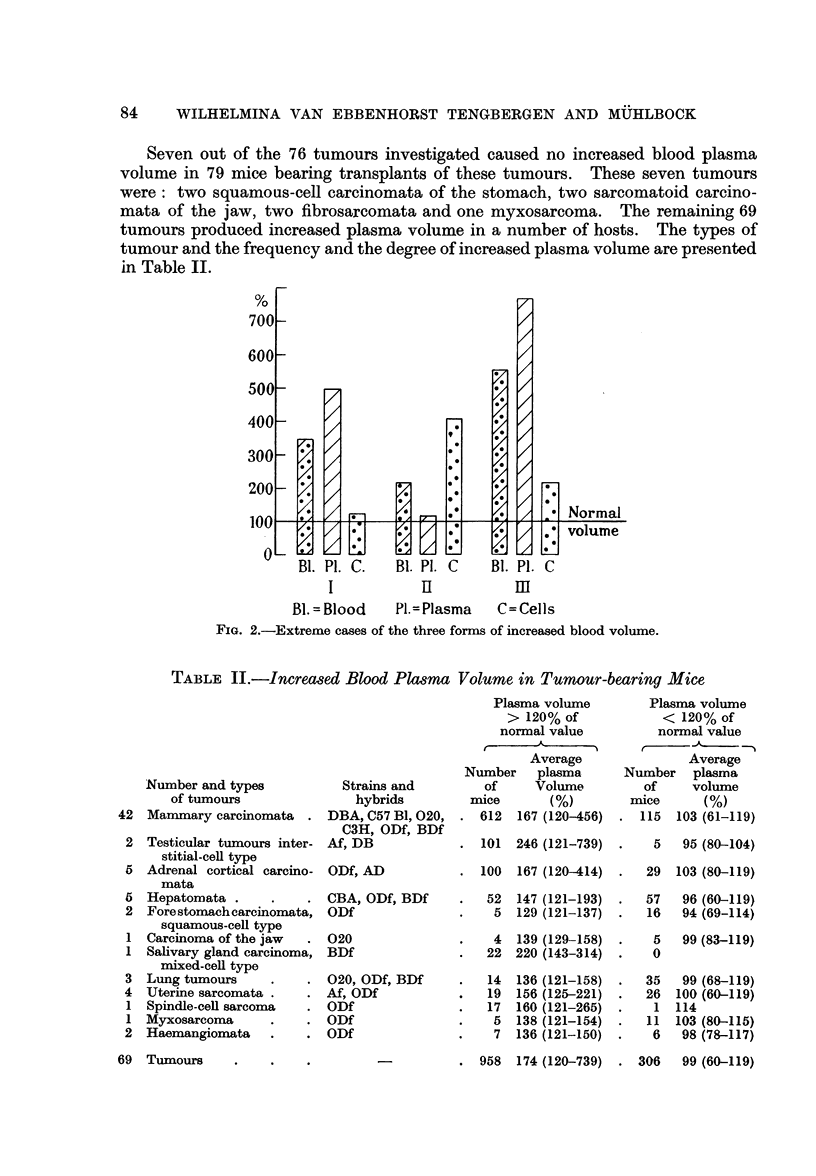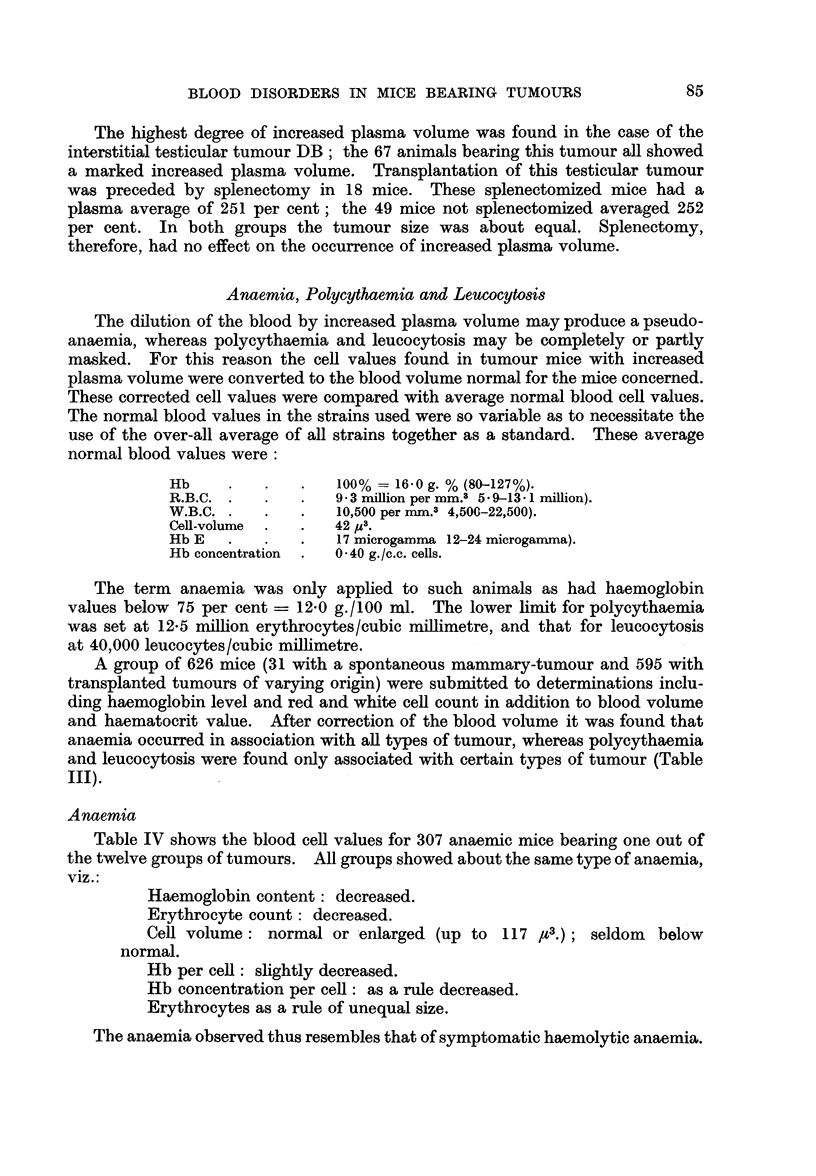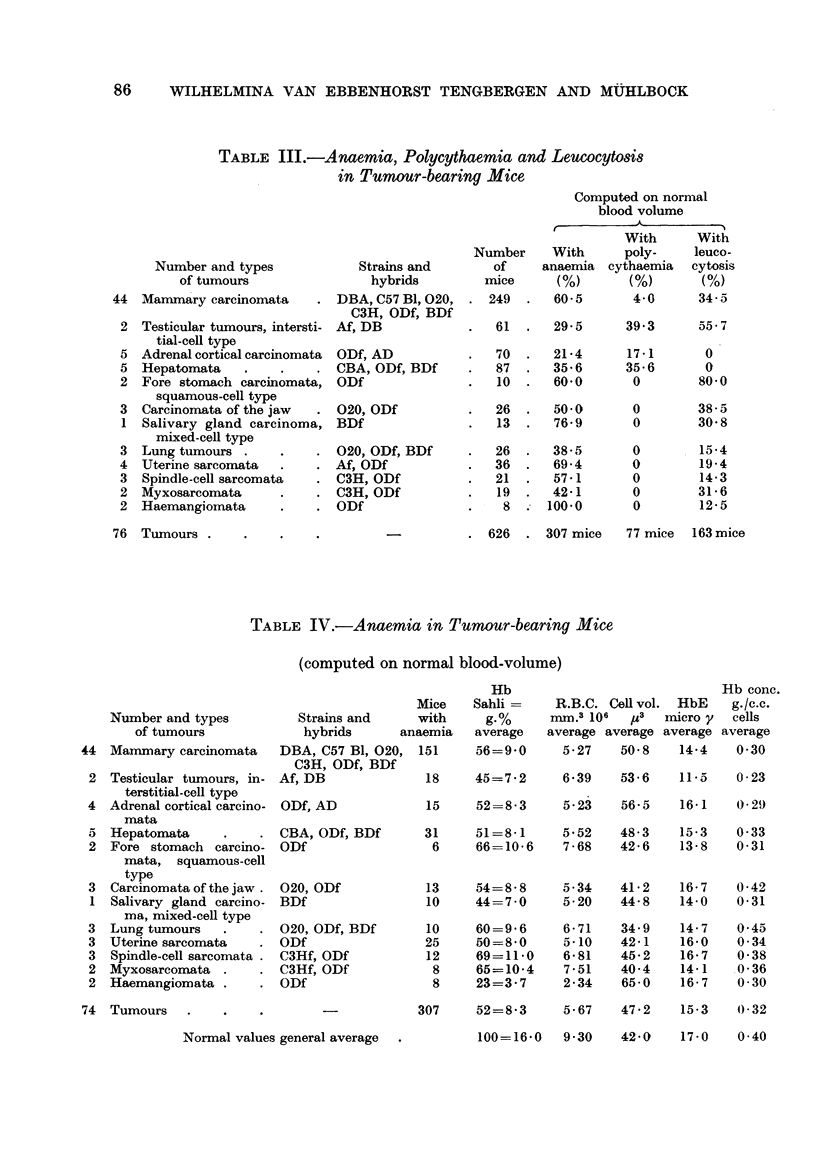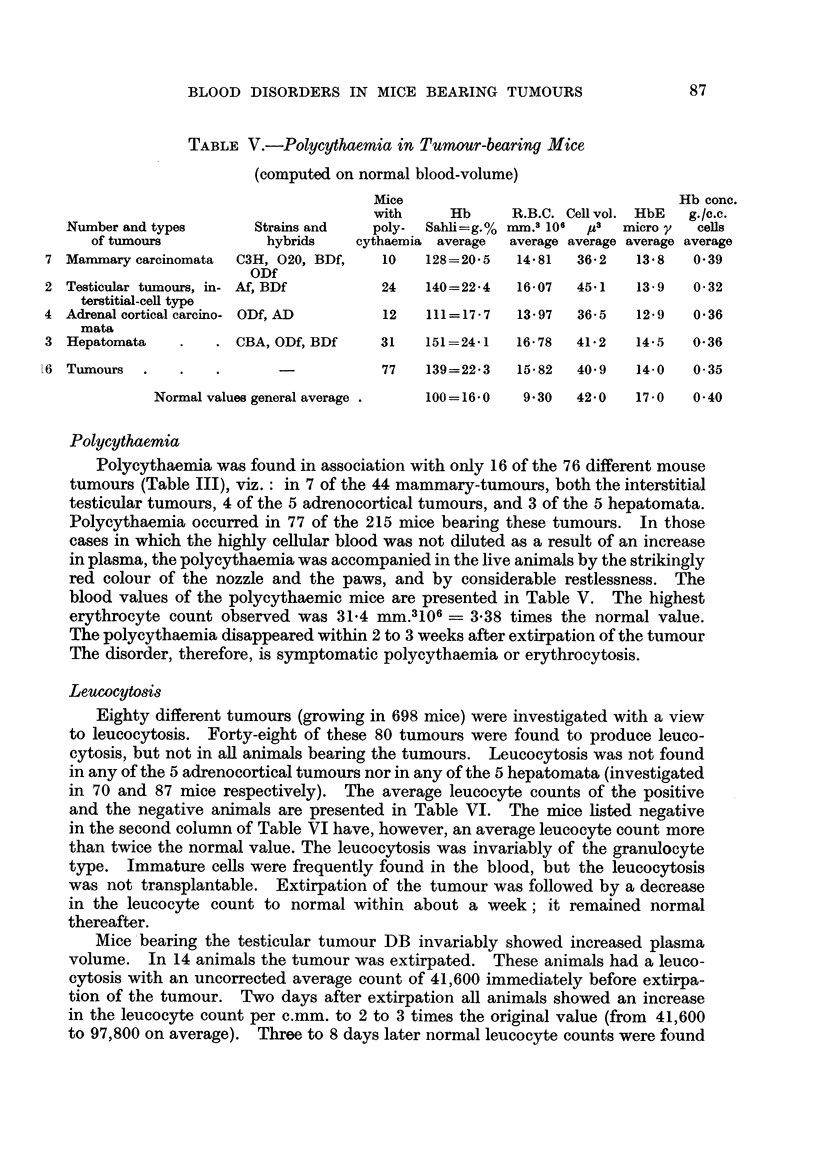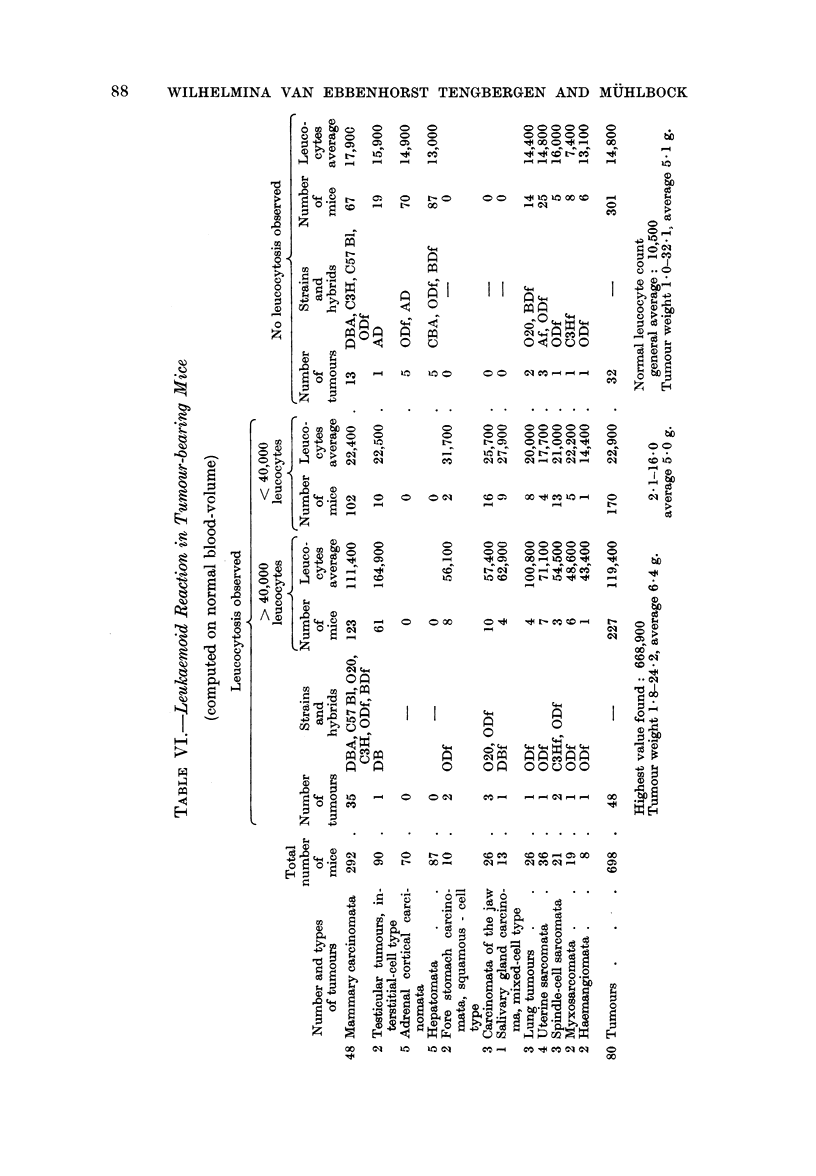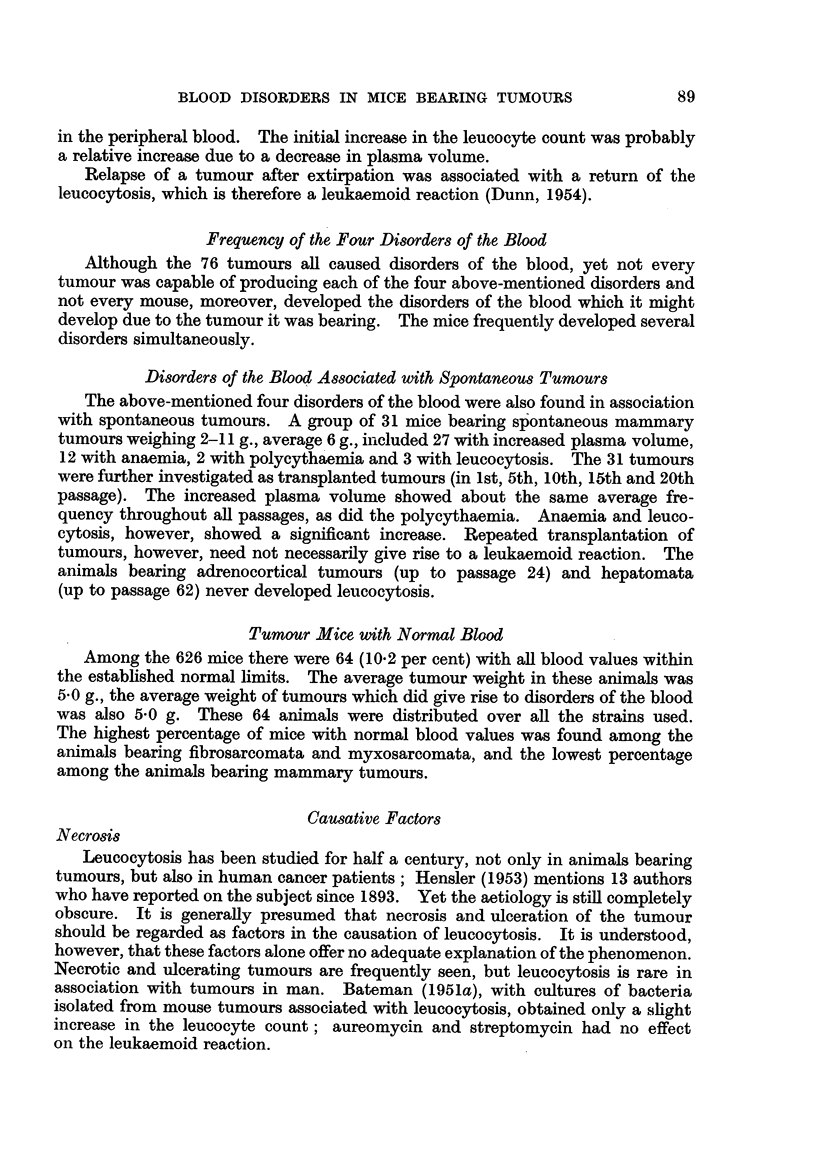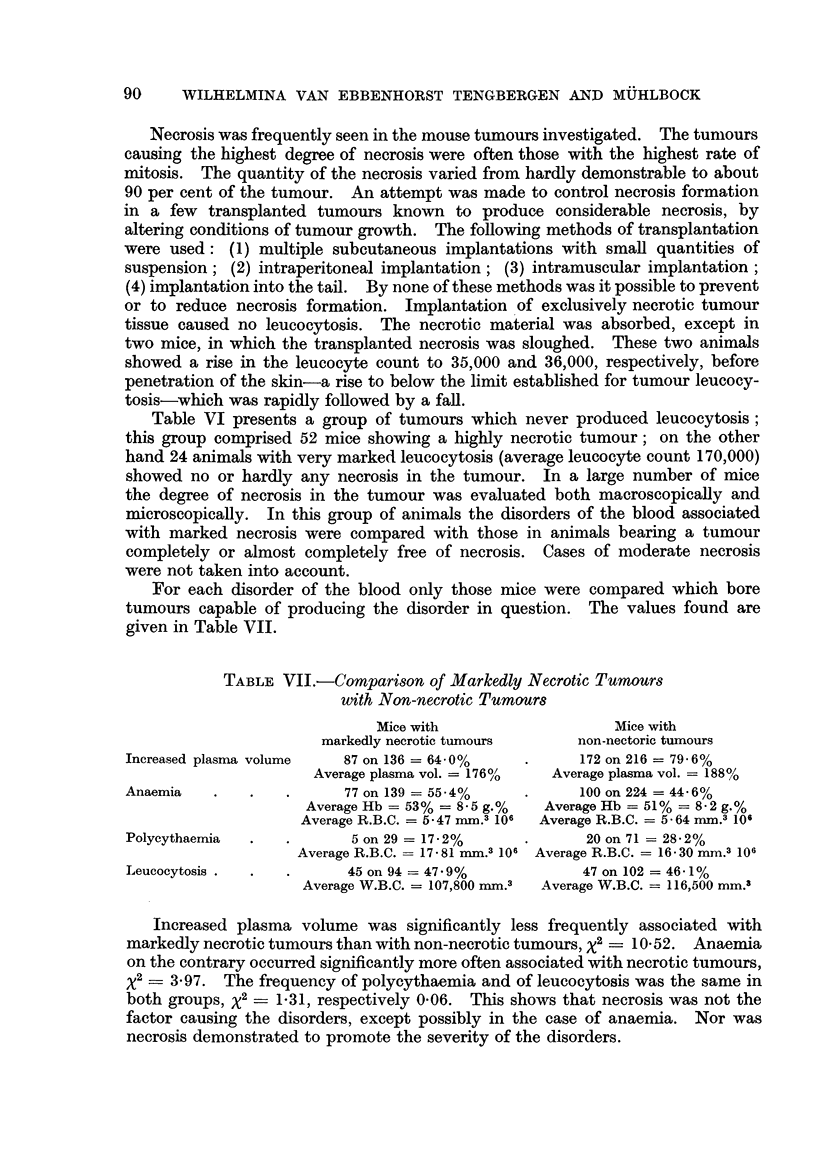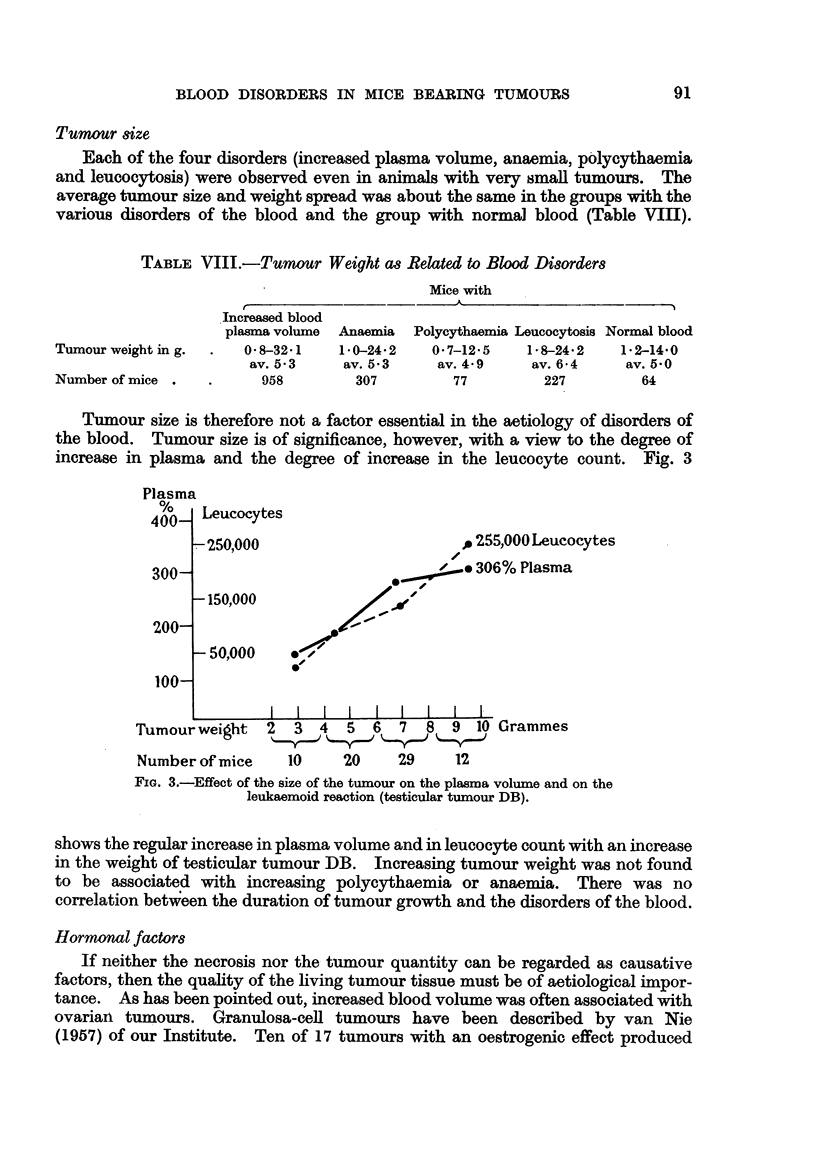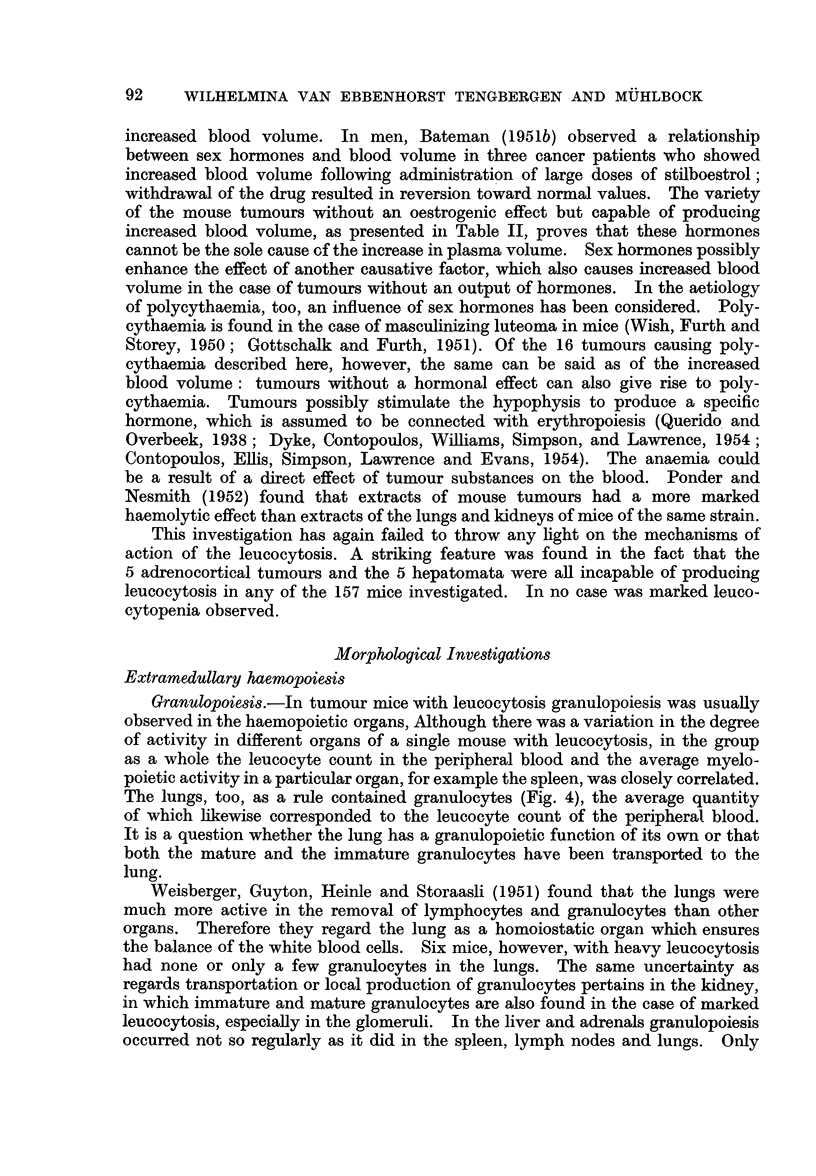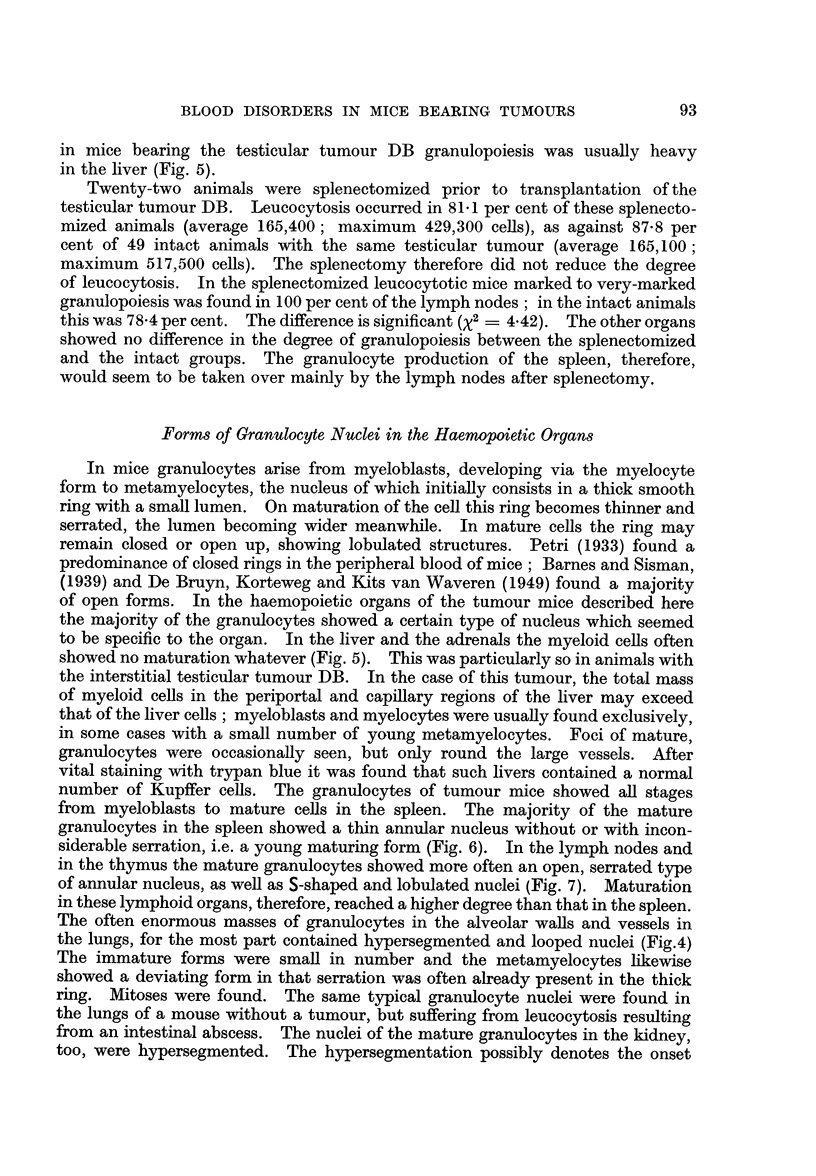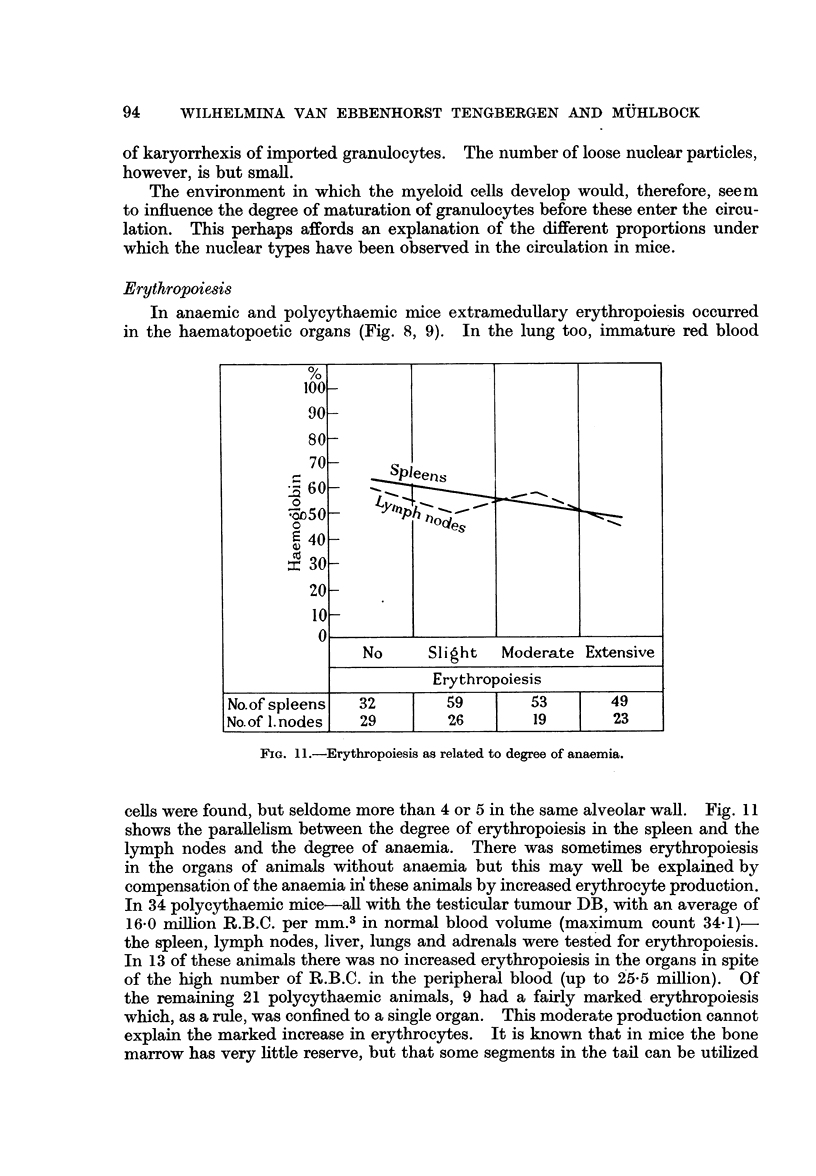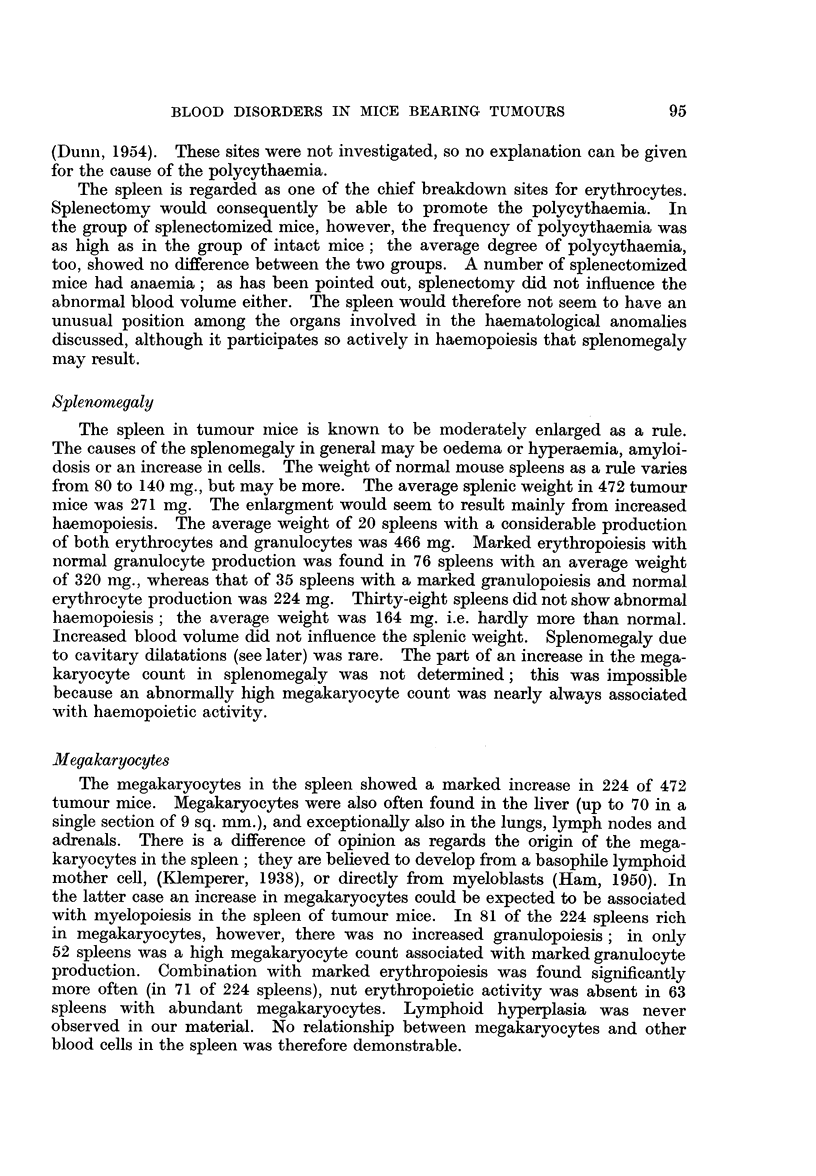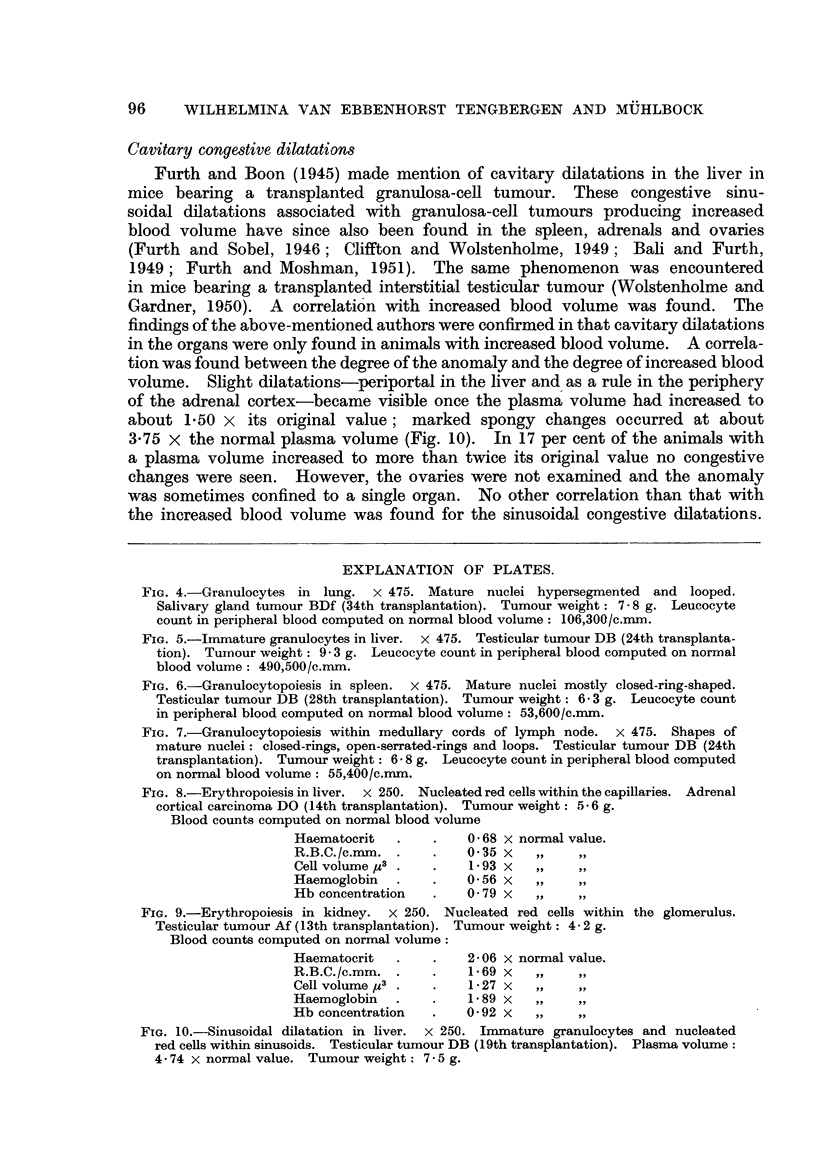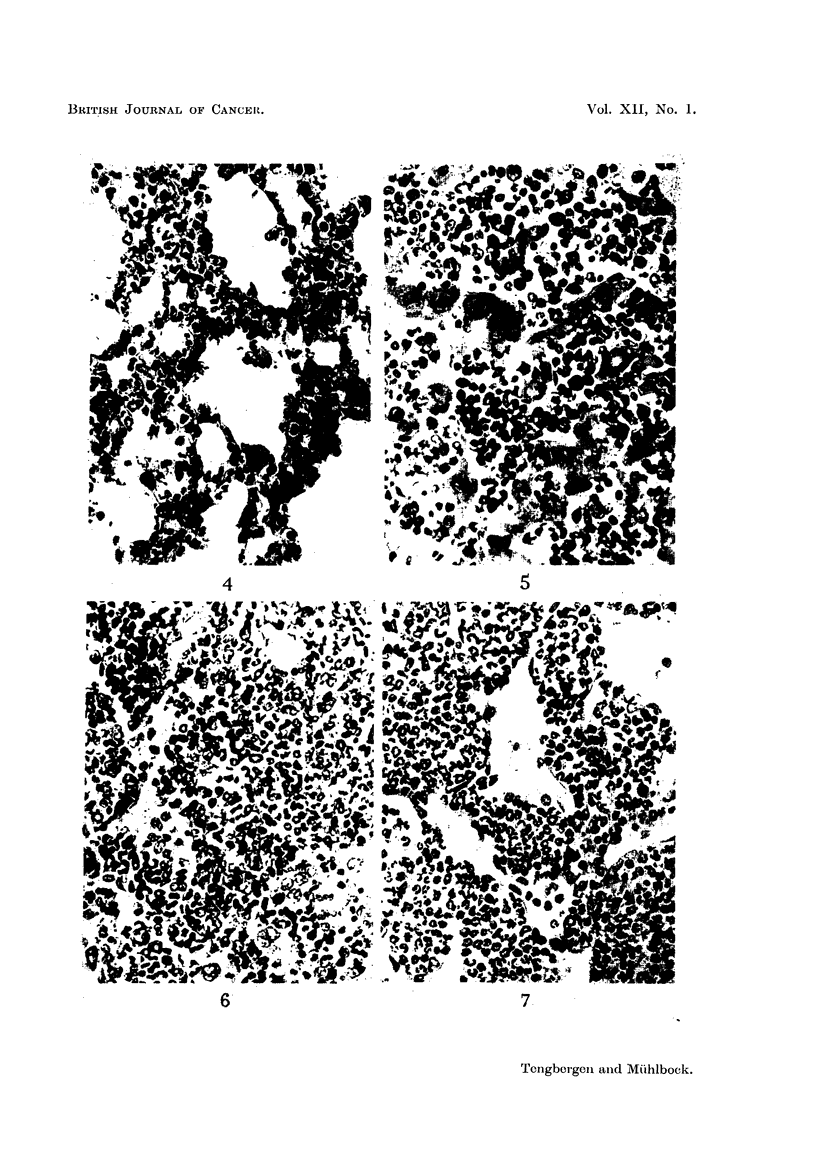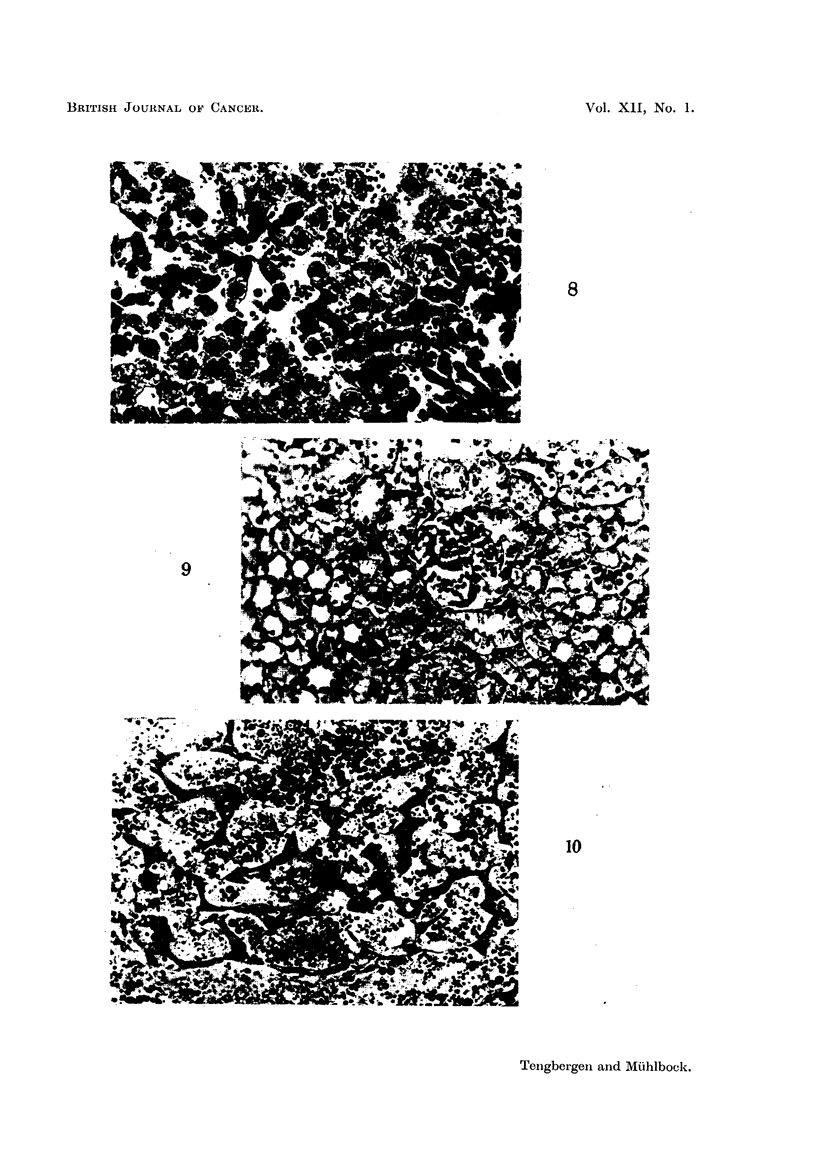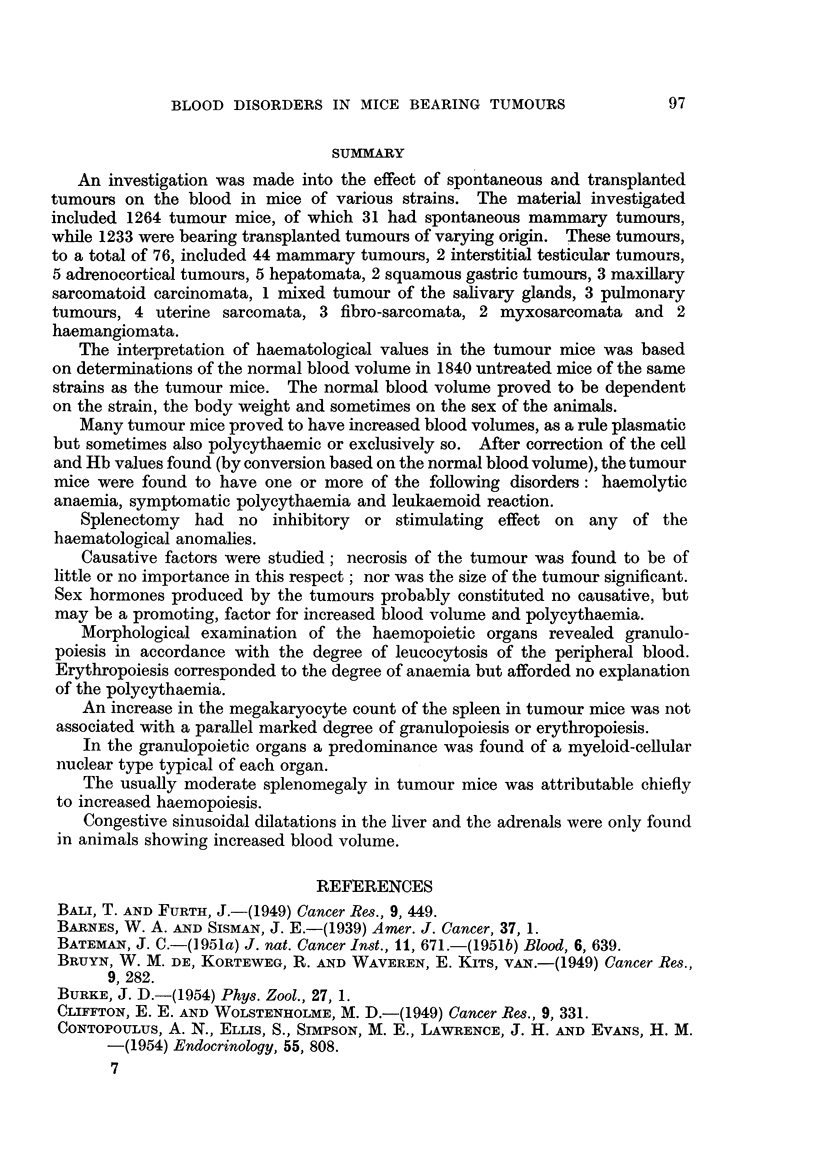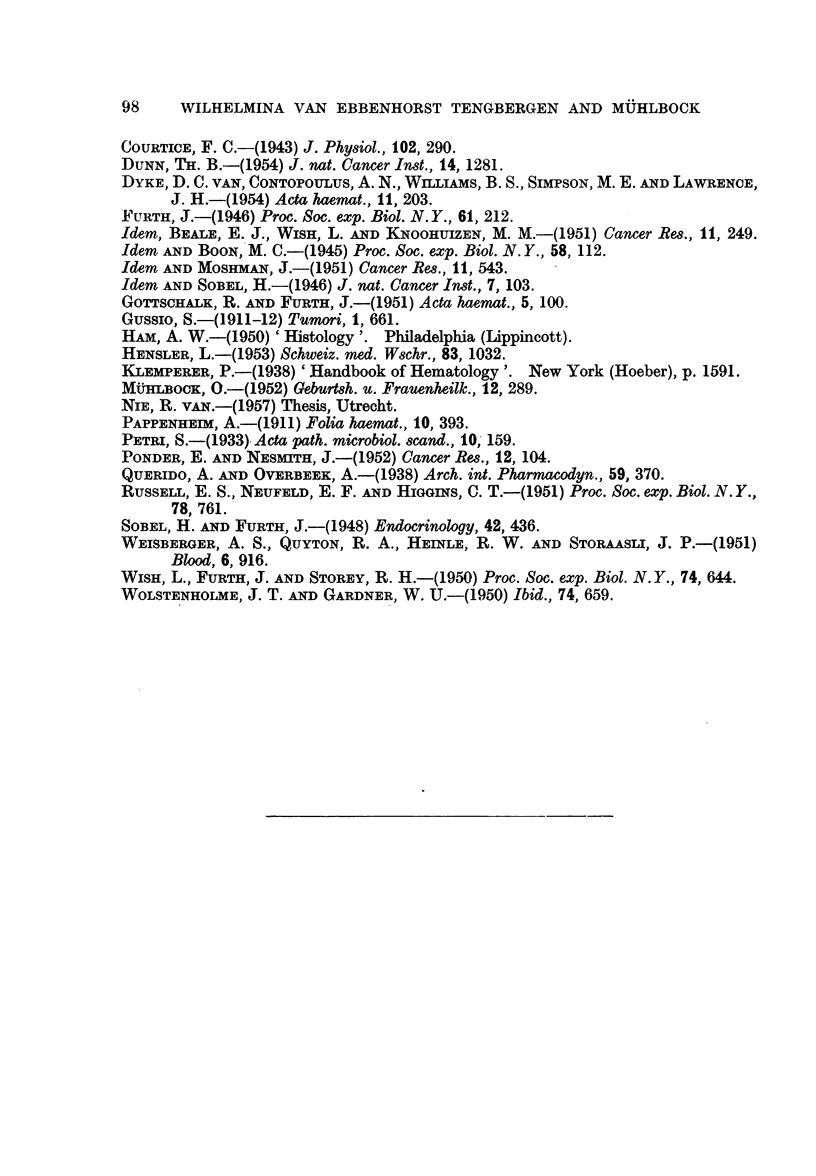# Disorders of the Blood in Mice Bearing Spontaneous or Transplanted Tumours

**DOI:** 10.1038/bjc.1958.12

**Published:** 1958-03

**Authors:** J. P. R. Wilhelmina, Tengbergen van Ebbenhorst, O. Mühlbock

## Abstract

**Images:**


### DISORDERS OF THE BLOOD IN MICE BEARING SPONTANEOUS OR TRANSPLANTED TUMOURS

WILHELMINA J. P. R. VAN EBBENHORST TENGBERGEN AND O. MÜHLBOCK

*From the Laboratory of the Antoni van Leeuwenhoekhuis, The Netherlands' Cancer Institute, Amsterdam, Holland*

Received for publication November 29, 1957

DISORDERS of the blood in mice bearing tumours have repeatedly been described. The first disorder to draw attention was leucocytosis; Pappenheim (1911) stated that leucocyte counts can reach very high values even in mice bearing small tumours. The first reports on increased blood volume in rats bearing sarcomata date back to about the same period (Gussio, 1911). It was not until 1946, however, that Furth and Sobel (1946) and other investigators, presented detailed reports on increased blood volume in mice bearing tumours, particularly ovarian tumours. It is surprising that anaemia in tumour-bearing animals received no special attention, whereas the literature comprises numerous reports on anaemia in cancer patients. Since 1950, polycythaemia has frequently been observed in mice bearing luteomata (Gottschalk and Furth, 1951; Wish, Furth and Storey, 1950; Furth and Moshman, 1951). This paper concerns an investigation into disorders of the blood in mice bearing spontaneous or transplanted tumours of varying origin. The aspects discussed are, in order of sequence: the blood volume in normal mice; increased blood volume, anaemia, polycythaemia and leucocytosis in tumour-bearing mice; the significance of necrosis and tumour size with a view to disorders of the blood; extra-medullary haemopoiesis; splenomegaly and cavitary congestive dilation in the organs.

### MATERIAL AND METHODS

The mouse strains used were DBA, C57 Bl, O20, C3H, A and CBA and the $F_1$ hybrids C57 Bl × DBAf (=BDf), O20 × DBAf (= ODf), and Af × DBAf (= Af Df). The 76 tumours used in the investigation can be divided into 12 types, viz.: mammary carcinomata, interstitial-cell testicular tumours, adrenocortical tumours, hepatomata, gastric squamous-cell carcinomata, carcinomata of the jaw, a mixed tumour of the salivary gland, pulmonary papillomata, uterine sarcomata, fibrosarcomata, myxosarcomata and haemangiomata. In view of their transplantability all these tumours, including those showing no infiltration or metastasis, are regarded as more or less malignant in mice. Method of tumour transplantation: subcutaneous, in exceptional cases intraperitoneal, intramuscular or into the tail using a suspension of finely-cut tumour tissue in a physiological saline solution.

Comparisons between the blood volume in normal and that in tumour-bearing animals were made by the decapitation method. Furth and Moshman (1951) state that exsanguination even without transfusion gives useful information. This simple method was used. In consequence only the blood from the large vessels was measured. Decapitation was done under light ether anaesthesia, and the blood flowing from the trunk and from the head was collected in a small, calibrated centrifuge tube containing 0·5 ml. citrate. The blood volume was read, and the tubes were then centrifuged in order to determine the plasma-cell ratio. The cell counts of the blood were made by the usual methods. The haemoglobin content was determined in a gauged Sahli haemometer. The blood was obtained from the tail without pre-warming.

For morphological examination tumours and organs were fixed in Heidenhain's " Susa " mixture. Sections were stained by haematoxylin-azophloxin staining, and blood and organic smears by Giemsa staining.

*The Blood Volume in Normal Mice*

In 1840 untreated mice belonging to the same pure and $F_1$-hybrid strains as the tumour mice under investigation, the blood volume from the large vessels varied from 2·2 to 6·0 ml./100 g. body weight. In animals weighing $\pm$ 25 g. the average volume was 4·16 per cent. The large vessels, therefore, probably contain less than 50 per cent of the total blood volume. The quantity of blood proved to be largely dependent on strain and body weight. In the majority of strains, moreover, a difference was found between the sexes : the females as a rule had more blood than the males (Table I).

TABLE I.—*Blood Volume in Untreated Female and Male Mice*

| | Body weight 20–25 g. | | | | Body weight 25–30 g. | | | |
|---|---|---|---|---|---|---|---|---|
| Strains and hybrids | Number of mice | ♀ ml. average | Number | ♂ ml. average | Number of mice | ♀ ml. average | Number | ♂ ml. average |
| DBA | 40 | 1·08 | 59 | 0·98 S | 16 | 1·33 | 20 | 1·23 S |
| C57 Bl | 6 | 0·95 | 20 | 0·84 S | 27 | 1·19 | 19 | 0·94 S |
| O20 | 53 | 0·98 | 70 | 0·75 S | 59 | 1·09 | 21 | 0·99 S |
| C3H | 42 | 1·06 | 34 | 1·00 | 26 | 1·19 | 28 | 1·16 |
| CBA | 15 | 0·97 | 6 | 0·97 | 37 | 1·10 | 27 | 1·07 |
| $F_1$BDf | 34 | 1·00 | 62 | 0·85 S | 10 | 1·12 | 60 | 1·02 |
| $F_1$ODf | 59 | 0·98 | 46 | 0·84 S | 20 | 1·09 | 44 | 0·99 S |

S = Difference between males and females is significant.

The age of the animals had no demonstrable effect on the blood volume, with the exception of the males of $F_1$ DBf, in which the blood volume determined later in life was relatively larger than that found in the young animals. Castration, oestrone treatment and presence of the mammary tumour agent failed to cause a change in blood volume. All strains showed a gradual increase in blood volume with an increase in body weight (Table I), but, if expressed in percentage of body weight, there was a relative decrease in the blood volume (Fig. 1).

Burke (1954) likewise found a percentual decrease in blood volume with an increase in body weight in man, opossum, rabbit, rat, sheep, horse, guinea-pig, hog and dog. Courtice (1943), who calculated the blood volume in rabbit, dog, goat and horse, found a similar relationship between blood volume and mass of tissue, chiefly muscle tissue.

The plasma-cell ratio was determined in 690 normal mice. The average haematocrit values in the various strains varied from 33·9 to 42·9 averaging

38·8. According to Wish, Furth and Storey (1950), the total blood volume in mice has a haematocrit value which is 0·88 times that of the venous blood alone. The haematocrit value of 38·8 found in the blood from the large vessels, therefore, would correspond with 44 in the venous blood, i.e. about as much as the value found by Russell, Neufeld and Higgins (1951) in 18 different mouse strains.

FIG. 1.—Blood volume in untreated mice. Percentage of body weight.

*The Blood Volume in Tumour-bearing Mice.*

Increased blood volume in mice bearing spontaneous and transplanted tumours has been repeatedly described in animals bearing ovarian tumours, (Furth and Sobel, 1946; Furth, 1946; Sobel and Furth, 1948; Bali and Furth, 1949; Cliffton and Wolstenholme, 1949; Furth and Moshman 1951; Mühlbock, 1952; van Nie, 1957) a transplanted testicular tumour (Wolstenholme and Gardner, 1950) and spontaneous mammary tumours (Furth and Moshman, 1951).

In our investigations blood volume determinations were made in a large number of mice bearing numerous different tumours. Many of these animals had a larger blood volume than untreated mice. According to Furth, Beale, Wish and Knoohuizen (1951), the tumour tissue in mice is relatively oligaemic as compared with the organic tissues. A mouse which has gained body weight due to a tumour, therefore, can be compared with a mouse which has gained weight as a result of normal growth (in which case the blood volume per 100 g. body weight is likewise decreased). The blood volume of tumour-bearing mice can therefore be compared with that of normal mice of the same strain, with the same body weight and of the same sex. The haematocrit value shows that the increased blood volume chiefly resulted from an excess of plasma. In a number of cases the cellular volume was also increased. The volume values for whole blood, plasma and cellular mass in untreated mice were regarded as 100 per cent. Values exceeding 120 per cent were regarded as above normal. Extreme examples of the three types of increased blood volume (plasmatic, polycythaemic and mixed) are presented in Fig. 2.

Seven out of the 76 tumours investigated caused no increased blood plasma volume in 79 mice bearing transplants of these tumours. These seven tumours were : two squamous-cell carcinomata of the stomach, two sarcomatoid carcinomata of the jaw, two fibrosarcomata and one myxosarcoma. The remaining 69 tumours produced increased plasma volume in a number of hosts. The types of tumour and the frequency and the degree of increased plasma volume are presented in Table II.

FIG. 2.—Extreme cases of the three forms of increased blood volume.

TABLE II.—*Increased Blood Plasma Volume in Tumour-bearing Mice*

| | Number and types of tumours | Strains and hybrids | Plasma volume > 120% of normal value: Number of mice | Plasma volume > 120% of normal value: Average plasma Volume (%) | Plasma volume < 120% of normal value: Number of mice | Plasma volume < 120% of normal value: Average plasma volume (%) |
|---|---|---|---|---|---|---|
| 42 | Mammary carcinomata | DBA, C57 Bl, O20, C3H, ODf, BDf | 612 | 167 (120–456) | 115 | 103 (61–119) |
| 2 | Testicular tumours interstitial-cell type | Af, DB | 101 | 246 (121–739) | 5 | 95 (80–104) |
| 5 | Adrenal cortical carcinomata | ODf, AD | 100 | 167 (120–414) | 29 | 103 (80–119) |
| 5 | Hepatomata | CBA, ODf, BDf | 52 | 147 (121–193) | 57 | 96 (60–119) |
| 2 | Forestomach carcinomata, squamous-cell type | ODf | 5 | 129 (121–137) | 16 | 94 (69–114) |
| 1 | Carcinoma of the jaw | O20 | 4 | 139 (129–158) | 5 | 99 (83–119) |
| 1 | Salivary gland carcinoma, mixed-cell type | BDf | 22 | 220 (143–314) | 0 | |
| 3 | Lung tumours | O20, ODf, BDf | 14 | 136 (121–158) | 35 | 99 (68–119) |
| 4 | Uterine sarcomata | Af, ODf | 19 | 156 (125–221) | 26 | 100 (60–119) |
| 1 | Spindle-cell sarcoma | ODf | 17 | 160 (121–265) | 1 | 114 |
| 1 | Myxosarcoma | ODf | 5 | 138 (121–154) | 11 | 103 (80–115) |
| 2 | Haemangiomata | ODf | 7 | 136 (121–150) | 6 | 98 (78–117) |
| 69 | Tumours | — | 958 | 174 (120–739) | 306 | 99 (60–119) |

The highest degree of increased plasma volume was found in the case of the interstitial testicular tumour DB ; the 67 animals bearing this tumour all showed a marked increased plasma volume. Transplantation of this testicular tumour was preceded by splenectomy in 18 mice. These splenectomized mice had a plasma average of 251 per cent ; the 49 mice not splenectomized averaged 252 per cent. In both groups the tumour size was about equal. Splenectomy, therefore, had no effect on the occurrence of increased plasma volume.

### *Anaemia, Polycythaemia and Leucocytosis*

The dilution of the blood by increased plasma volume may produce a pseudo-anaemia, whereas polycythaemia and leucocytosis may be completely or partly masked. For this reason the cell values found in tumour mice with increased plasma volume were converted to the blood volume normal for the mice concerned. These corrected cell values were compared with average normal blood cell values. The normal blood values in the strains used were so variable as to necessitate the use of the over-all average of all strains together as a standard. These average normal blood values were :

| | |
|---|---|
| Hb . . . . | 100% = 16·0 g. % (80–127%). |
| R.B.C. . . . | 9·3 million per $mm.^3$ 5·9–13·1 million). |
| W.B.C. . . . | 10,500 per $mm.^3$ 4,500–22,500). |
| Cell-volume . . | 42 $\mu^3$. |
| Hb E . . . | 17 microgamma 12–24 microgamma). |
| Hb concentration . | 0·40 g./c.c. cells. |

The term anaemia was only applied to such animals as had haemoglobin values below 75 per cent = 12·0 g./100 ml. The lower limit for polycythaemia was set at 12·5 million erythrocytes/cubic millimetre, and that for leucocytosis at 40,000 leucocytes/cubic millimetre.

A group of 626 mice (31 with a spontaneous mammary-tumour and 595 with transplanted tumours of varying origin) were submitted to determinations including haemoglobin level and red and white cell count in addition to blood volume and haematocrit value. After correction of the blood volume it was found that anaemia occurred in association with all types of tumour, whereas polycythaemia and leucocytosis were found only associated with certain types of tumour (Table III).

#### *Anaemia*

Table IV shows the blood cell values for 307 anaemic mice bearing one out of the twelve groups of tumours. All groups showed about the same type of anaemia, viz.:

Haemoglobin content : decreased.
Erythrocyte count : decreased.
Cell volume : normal or enlarged (up to 117 $\mu^3$.) ; seldom below normal.
Hb per cell : slightly decreased.
Hb concentration per cell : as a rule decreased.
Erythrocytes as a rule of unequal size.

The anaemia observed thus resembles that of symptomatic haemolytic anaemia.

TABLE III.—*Anaemia, Polycythaemia and Leucocytosis in Tumour-bearing Mice*

| | Number and types of tumours | Strains and hybrids | Number of mice | Computed on normal blood volume: With anaemia (%) | With polycythaemia (%) | With leucocytosis (%) |
|---|---|---|---|---|---|---|
| 44 | Mammary carcinomata | DBA, C57 Bl, O20, C3H, ODf, BDf | 249 | 60·5 | 4·0 | 34·5 |
| 2 | Testicular tumours, interstitial-cell type | Af, DB | 61 | 29·5 | 39·3 | 55·7 |
| 5 | Adrenal cortical carcinomata | ODf, AD | 70 | 21·4 | 17·1 | 0 |
| 5 | Hepatomata | CBA, ODf, BDf | 87 | 35·6 | 35·6 | 0 |
| 2 | Fore stomach carcinomata, squamous-cell type | ODf | 10 | 60·0 | 0 | 80·0 |
| 3 | Carcinomata of the jaw | O20, ODf | 26 | 50·0 | 0 | 38·5 |
| 1 | Salivary gland carcinoma, mixed-cell type | BDf | 13 | 76·9 | 0 | 30·8 |
| 3 | Lung tumours | O20, ODf, BDf | 26 | 38·5 | 0 | 15·4 |
| 4 | Uterine sarcomata | Af, ODf | 36 | 69·4 | 0 | 19·4 |
| 3 | Spindle-cell sarcomata | C3H, ODf | 21 | 57·1 | 0 | 14·3 |
| 2 | Myxosarcomata | C3H, ODf | 19 | 42·1 | 0 | 31·6 |
| 2 | Haemangiomata | ODf | 8 | 100·0 | 0 | 12·5 |
| 76 | Tumours | — | 626 | 307 mice | 77 mice | 163 mice |

TABLE IV.—*Anaemia in Tumour-bearing Mice*

(computed on normal blood-volume)

| | Number and types of tumours | Strains and hybrids | Mice with anaemia | Hb Sahli = g.% average | R.B.C. mm.³ 10⁶ average | Cell vol. $\mu^3$ average | HbE micro $\gamma$ average | Hb conc. g./c.c. cells average |
|---|---|---|---|---|---|---|---|---|
| 44 | Mammary carcinomata | DBA, C57 Bl, O20, C3H, ODf, BDf | 151 | 56=9·0 | 5·27 | 50·8 | 14·4 | 0·30 |
| 2 | Testicular tumours, interstitial-cell type | Af, DB | 18 | 45=7·2 | 6·39 | 53·6 | 11·5 | 0·23 |
| 4 | Adrenal cortical carcinomata | ODf, AD | 15 | 52=8·3 | 5·23 | 56·5 | 16·1 | 0·29 |
| 5 | Hepatomata | CBA, ODf, BDf | 31 | 51=8·1 | 5·52 | 48·3 | 15·3 | 0·33 |
| 2 | Fore stomach carcinomata, squamous-cell type | ODf | 6 | 66=10·6 | 7·68 | 42·6 | 13·8 | 0·31 |
| 3 | Carcinomata of the jaw | O20, ODf | 13 | 54=8·8 | 5·34 | 41·2 | 16·7 | 0·42 |
| 1 | Salivary gland carcinoma, mixed-cell type | BDf | 10 | 44=7·0 | 5·20 | 44·8 | 14·0 | 0·31 |
| 3 | Lung tumours | O20, ODf, BDf | 10 | 60=9·6 | 6·71 | 34·9 | 14·7 | 0·45 |
| 3 | Uterine sarcomata | ODf | 25 | 50=8·0 | 5·10 | 42·1 | 16·0 | 0·34 |
| 3 | Spindle-cell sarcomata | C3Hf, ODf | 12 | 69=11·0 | 6·81 | 45·2 | 16·7 | 0·38 |
| 2 | Myxosarcomata | C3Hf, ODf | 8 | 65=10·4 | 7·51 | 40·4 | 14·1 | 0·36 |
| 2 | Haemangiomata | ODf | 8 | 23=3·7 | 2·34 | 65·0 | 16·7 | 0·30 |
| 74 | Tumours | — | 307 | 52=8·3 | 5·67 | 47·2 | 15·3 | 0·32 |
| | Normal values general average | | | 100=16·0 | 9·30 | 42·0 | 17·0 | 0·40 |

TABLE V.—*Polycythaemia in Tumour-bearing Mice*

(computed on normal blood-volume)

| | Number and types of tumours | Strains and hybrids | Mice with poly-cythaemia | Hb Sahli=g.% average | R.B.C. mm.³ 10⁶ average | Cell vol. μ³ average | HbE micro γ average | Hb conc. g./c.c. cells average |
|---|---|---|---|---|---|---|---|---|
| 7 | Mammary carcinomata | C3H, O20, BDf, ODf | 10 | 128=20·5 | 14·81 | 36·2 | 13·8 | 0·39 |
| 2 | Testicular tumours, interstitial-cell type | Af, BDf | 24 | 140=22·4 | 16·07 | 45·1 | 13·9 | 0·32 |
| 4 | Adrenal cortical carcinomata | ODf, AD | 12 | 111=17·7 | 13·97 | 36·5 | 12·9 | 0·36 |
| 3 | Hepatomata . . . | CBA, ODf, BDf | 31 | 151=24·1 | 16·78 | 41·2 | 14·5 | 0·36 |
| 16 | Tumours . . . | — | 77 | 139=22·3 | 15·82 | 40·9 | 14·0 | 0·35 |
| | Normal values general average . | | | 100=16·0 | 9·30 | 42·0 | 17·0 | 0·40 |

*Polycythaemia*

Polycythaemia was found in association with only 16 of the 76 different mouse tumours (Table III), viz.: in 7 of the 44 mammary-tumours, both the interstitial testicular tumours, 4 of the 5 adrenocortical tumours, and 3 of the 5 hepatomata. Polycythaemia occurred in 77 of the 215 mice bearing these tumours. In those cases in which the highly cellular blood was not diluted as a result of an increase in plasma, the polycythaemia was accompanied in the live animals by the strikingly red colour of the nozzle and the paws, and by considerable restlessness. The blood values of the polycythaemic mice are presented in Table V. The highest erythrocyte count observed was 31·4 $mm.^3 10^6$ = 3·38 times the normal value. The polycythaemia disappeared within 2 to 3 weeks after extirpation of the tumour The disorder, therefore, is symptomatic polycythaemia or erythrocytosis.

*Leucocytosis*

Eighty different tumours (growing in 698 mice) were investigated with a view to leucocytosis. Forty-eight of these 80 tumours were found to produce leucocytosis, but not in all animals bearing the tumours. Leucocytosis was not found in any of the 5 adrenocortical tumours nor in any of the 5 hepatomata (investigated in 70 and 87 mice respectively). The average leucocyte counts of the positive and the negative animals are presented in Table VI. The mice listed negative in the second column of Table VI have, however, an average leucocyte count more than twice the normal value. The leucocytosis was invariably of the granulocyte type. Immature cells were frequently found in the blood, but the leucocytosis was not transplantable. Extirpation of the tumour was followed by a decrease in the leucocyte count to normal within about a week; it remained normal thereafter.

Mice bearing the testicular tumour DB invariably showed increased plasma volume. In 14 animals the tumour was extirpated. These animals had a leucocytosis with an uncorrected average count of 41,600 immediately before extirpation of the tumour. Two days after extirpation all animals showed an increase in the leucocyte count per c.mm. to 2 to 3 times the original value (from 41,600 to 97,800 on average). Three to 8 days later normal leucocyte counts were found

TABLE VI.—*Leukaemoid Reaction in Tumour-bearing Mice*

(computed on normal blood-volume)

| Number and types of tumours | Total number of mice | Leucocytosis observed: Number of tumours | Strains and hybrids | > 40,000 leucocytes: Number of mice | Leucocytes average | < 40,000 leucocytes: Number of mice | Leucocytes average | No leucocytosis observed: Number of tumours | Strains and hybrids | Number of mice | Leucocytes average |
|---|---|---|---|---|---|---|---|---|---|---|---|
| 48 Mammary carcinomata | 292 | 35 | DBA, C57 Bl, O20, C3H, ODf, BDf | 123 | 111,400 | 102 | 22,400 | 13 | DBA, C3H, C57 Bl, ODf | 67 | 17,90C |
| 2 Testicular tumours, interstitial-cell type | 90 | 1 | DB | 61 | 164,900 | 10 | 22,500 | 1 | AD | 19 | 15,900 |
| 5 Adrenal cortical carcinomata | 70 | 0 | — | 0 | | 0 | | 5 | ODf, AD | 70 | 14,900 |
| 5 Hepatomata | 87 | 0 | — | 0 | | 0 | | 5 | CBA, ODf, BDf | 87 | 13,000 |
| 2 Fore stomach carcinomata, squamous - cell type | 10 | 2 | ODf | 8 | 56,100 | 2 | 31,700 | 0 | — | 0 | |
| 3 Carcinomata of the jaw | 26 | 3 | O20, ODf | 10 | 57,400 | 16 | 25,700 | 0 | — | 0 | |
| 1 Salivary gland carcinoma, mixed-cell type | 13 | 1 | DBf | 4 | 62,900 | 9 | 27,900 | 0 | — | 0 | |
| 3 Lung tumours | 26 | 1 | ODf | 4 | 100,800 | 8 | 20,000 | 2 | O20, BDf | 14 | 14,400 |
| 4 Uterine sarcomata | 36 | 1 | ODf | 7 | 71,100 | 4 | 17,700 | 3 | Af, ODf | 25 | 14,800 |
| 3 Spindle-cell sarcomata | 21 | 2 | C3Hf, ODf | 3 | 54,500 | 13 | 21,000 | 1 | ODf | 5 | 16,000 |
| 2 Myxosarcomata | 19 | 1 | ODf | 6 | 48,600 | 5 | 22,200 | 1 | C3Hf | 8 | 7,400 |
| 2 Haemangiomata | 8 | 1 | ODf | 1 | 43,400 | 1 | 14,400 | 1 | ODf | 6 | 13,100 |
| 80 Tumours | 698 | 48 | — | 227 | 119,400 | 170 | 22,900 | 32 | — | 301 | 14,800 |

> 40,000 leucocytes: Highest value found : 668,900; Tumour weight 1·8–24·2, average 6·4 g.

< 40,000 leucocytes: Tumour weight 2·1–16·0, average 5·0 g.

No leucocytosis observed: Normal leucocyte count general average : 10,500; Tumour weight 1·0–32·1, average 5·1 g.

in the peripheral blood. The initial increase in the leucocyte count was probably a relative increase due to a decrease in plasma volume.

Relapse of a tumour after extirpation was associated with a return of the leucocytosis, which is therefore a leukaemoid reaction (Dunn, 1954).

*Frequency of the Four Disorders of the Blood*

Although the 76 tumours all caused disorders of the blood, yet not every tumour was capable of producing each of the four above-mentioned disorders and not every mouse, moreover, developed the disorders of the blood which it might develop due to the tumour it was bearing. The mice frequently developed several disorders simultaneously.

*Disorders of the Blood Associated with Spontaneous Tumours*

The above-mentioned four disorders of the blood were also found in association with spontaneous tumours. A group of 31 mice bearing spontaneous mammary tumours weighing 2–11 g., average 6 g., included 27 with increased plasma volume, 12 with anaemia, 2 with polycythaemia and 3 with leucocytosis. The 31 tumours were further investigated as transplanted tumours (in 1st, 5th, 10th, 15th and 20th passage). The increased plasma volume showed about the same average frequency throughout all passages, as did the polycythaemia. Anaemia and leucocytosis, however, showed a significant increase. Repeated transplantation of tumours, however, need not necessarily give rise to a leukaemoid reaction. The animals bearing adrenocortical tumours (up to passage 24) and hepatomata (up to passage 62) never developed leucocytosis.

*Tumour Mice with Normal Blood*

Among the 626 mice there were 64 (10·2 per cent) with all blood values within the established normal limits. The average tumour weight in these animals was 5·0 g., the average weight of tumours which did give rise to disorders of the blood was also 5·0 g. These 64 animals were distributed over all the strains used. The highest percentage of mice with normal blood values was found among the animals bearing fibrosarcomata and myxosarcomata, and the lowest percentage among the animals bearing mammary tumours.

*Causative Factors*

*Necrosis*

Leucocytosis has been studied for half a century, not only in animals bearing tumours, but also in human cancer patients ; Hensler (1953) mentions 13 authors who have reported on the subject since 1893. Yet the aetiology is still completely obscure. It is generally presumed that necrosis and ulceration of the tumour should be regarded as factors in the causation of leucocytosis. It is understood, however, that these factors alone offer no adequate explanation of the phenomenon. Necrotic and ulcerating tumours are frequently seen, but leucocytosis is rare in association with tumours in man. Bateman (1951*a*), with cultures of bacteria isolated from mouse tumours associated with leucocytosis, obtained only a slight increase in the leucocyte count ; aureomycin and streptomycin had no effect on the leukaemoid reaction.

Necrosis was frequently seen in the mouse tumours investigated. The tumours causing the highest degree of necrosis were often those with the highest rate of mitosis. The quantity of the necrosis varied from hardly demonstrable to about 90 per cent of the tumour. An attempt was made to control necrosis formation in a few transplanted tumours known to produce considerable necrosis, by altering conditions of tumour growth. The following methods of transplantation were used: (1) multiple subcutaneous implantations with small quantities of suspension; (2) intraperitoneal implantation; (3) intramuscular implantation; (4) implantation into the tail. By none of these methods was it possible to prevent or to reduce necrosis formation. Implantation of exclusively necrotic tumour tissue caused no leucocytosis. The necrotic material was absorbed, except in two mice, in which the transplanted necrosis was sloughed. These two animals showed a rise in the leucocyte count to 35,000 and 36,000, respectively, before penetration of the skin—a rise to below the limit established for tumour leucocytosis—which was rapidly followed by a fall.

Table VI presents a group of tumours which never produced leucocytosis; this group comprised 52 mice showing a highly necrotic tumour; on the other hand 24 animals with very marked leucocytosis (average leucocyte count 170,000) showed no or hardly any necrosis in the tumour. In a large number of mice the degree of necrosis in the tumour was evaluated both macroscopically and microscopically. In this group of animals the disorders of the blood associated with marked necrosis were compared with those in animals bearing a tumour completely or almost completely free of necrosis. Cases of moderate necrosis were not taken into account.

For each disorder of the blood only those mice were compared which bore tumours capable of producing the disorder in question. The values found are given in Table VII.

TABLE VII.—*Comparison of Markedly Necrotic Tumours with Non-necrotic Tumours*

| | Mice with markedly necrotic tumours | Mice with non-nectoric tumours |
|---|---|---|
| Increased plasma volume | 87 on 136 = 64·0%<br>Average plasma vol. = 176% | 172 on 216 = 79·6%<br>Average plasma vol. = 188% |
| Anaemia | 77 on 139 = 55·4%<br>Average Hb = 53% = 8·5 g.%<br>Average R.B.C. = 5·47 mm.³ 10⁶ | 100 on 224 = 44·6%<br>Average Hb = 51% = 8·2 g.%<br>Average R.B.C. = 5·64 mm.³ 10⁶ |
| Polycythaemia | 5 on 29 = 17·2%<br>Average R.B.C. = 17·81 mm.³ 10⁶ | 20 on 71 = 28·2%<br>Average R.B.C. = 16·30 mm.³ 10⁶ |
| Leucocytosis | 45 on 94 = 47·9%<br>Average W.B.C. = 107,800 mm.³ | 47 on 102 = 46·1%<br>Average W.B.C. = 116,500 mm.³ |

Increased plasma volume was significantly less frequently associated with markedly necrotic tumours than with non-necrotic tumours, $\chi^2 = 10{\cdot}52$. Anaemia on the contrary occurred significantly more often associated with necrotic tumours, $\chi^2 = 3{\cdot}97$. The frequency of polycythaemia and of leucocytosis was the same in both groups, $\chi^2 = 1{\cdot}31$, respectively 0·06. This shows that necrosis was not the factor causing the disorders, except possibly in the case of anaemia. Nor was necrosis demonstrated to promote the severity of the disorders.

*Tumour size*

Each of the four disorders (increased plasma volume, anaemia, polycythaemia and leucocytosis) were observed even in animals with very small tumours. The average tumour size and weight spread was about the same in the groups with the various disorders of the blood and the group with normal blood (Table VIII).

TABLE VIII.—*Tumour Weight as Related to Blood Disorders*

| | Mice with | | | | |
|---|---|---|---|---|---|
| | Increased blood plasma volume | Anaemia | Polycythaemia | Leucocytosis | Normal blood |
| Tumour weight in g. . | 0·8–32·1 av. 5·3 | 1·0–24·2 av. 5·3 | 0·7–12·5 av. 4·9 | 1·8–24·2 av. 6·4 | 1·2–14·0 av. 5·0 |
| Number of mice . . | 958 | 307 | 77 | 227 | 64 |

Tumour size is therefore not a factor essential in the aetiology of disorders of the blood. Tumour size is of significance, however, with a view to the degree of increase in plasma and the degree of increase in the leucocyte count. Fig. 3

FIG. 3.—Effect of the size of the tumour on the plasma volume and on the leukaemoid reaction (testicular tumour DB).

shows the regular increase in plasma volume and in leucocyte count with an increase in the weight of testicular tumour DB. Increasing tumour weight was not found to be associated with increasing polycythaemia or anaemia. There was no correlation between the duration of tumour growth and the disorders of the blood.

*Hormonal factors*

If neither the necrosis nor the tumour quantity can be regarded as causative factors, then the quality of the living tumour tissue must be of aetiological importance. As has been pointed out, increased blood volume was often associated with ovarian tumours. Granulosa-cell tumours have been described by van Nie (1957) of our Institute. Ten of 17 tumours with an oestrogenic effect produced increased blood volume. In men, Bateman (1951*b*) observed a relationship between sex hormones and blood volume in three cancer patients who showed increased blood volume following administration of large doses of stilboestrol; withdrawal of the drug resulted in reversion toward normal values. The variety of the mouse tumours without an oestrogenic effect but capable of producing increased blood volume, as presented in Table II, proves that these hormones cannot be the sole cause of the increase in plasma volume. Sex hormones possibly enhance the effect of another causative factor, which also causes increased blood volume in the case of tumours without an output of hormones. In the aetiology of polycythaemia, too, an influence of sex hormones has been considered. Polycythaemia is found in the case of masculinizing luteoma in mice (Wish, Furth and Storey, 1950; Gottschalk and Furth, 1951). Of the 16 tumours causing polycythaemia described here, however, the same can be said as of the increased blood volume: tumours without a hormonal effect can also give rise to polycythaemia. Tumours possibly stimulate the hypophysis to produce a specific hormone, which is assumed to be connected with erythropoiesis (Querido and Overbeek, 1938; Dyke, Contopoulos, Williams, Simpson, and Lawrence, 1954; Contopoulos, Ellis, Simpson, Lawrence and Evans, 1954). The anaemia could be a result of a direct effect of tumour substances on the blood. Ponder and Nesmith (1952) found that extracts of mouse tumours had a more marked haemolytic effect than extracts of the lungs and kidneys of mice of the same strain.

This investigation has again failed to throw any light on the mechanisms of action of the leucocytosis. A striking feature was found in the fact that the 5 adrenocortical tumours and the 5 hepatomata were all incapable of producing leucocytosis in any of the 157 mice investigated. In no case was marked leucocytopenia observed.

### *Morphological Investigations*

### *Extramedullary haemopoiesis*

*Granulopoiesis.*—In tumour mice with leucocytosis granulopoiesis was usually observed in the haemopoietic organs, Although there was a variation in the degree of activity in different organs of a single mouse with leucocytosis, in the group as a whole the leucocyte count in the peripheral blood and the average myelopoietic activity in a particular organ, for example the spleen, was closely correlated. The lungs, too, as a rule contained granulocytes (Fig. 4), the average quantity of which likewise corresponded to the leucocyte count of the peripheral blood. It is a question whether the lung has a granulopoietic function of its own or that both the mature and the immature granulocytes have been transported to the lung.

Weisberger, Guyton, Heinle and Storaasli (1951) found that the lungs were much more active in the removal of lymphocytes and granulocytes than other organs. Therefore they regard the lung as a homoiostatic organ which ensures the balance of the white blood cells. Six mice, however, with heavy leucocytosis had none or only a few granulocytes in the lungs. The same uncertainty as regards transportation or local production of granulocytes pertains in the kidney, in which immature and mature granulocytes are also found in the case of marked leucocytosis, especially in the glomeruli. In the liver and adrenals granulopoiesis occurred not so regularly as it did in the spleen, lymph nodes and lungs. Only in mice bearing the testicular tumour DB granulopoiesis was usually heavy in the liver (Fig. 5).

Twenty-two animals were splenectomized prior to transplantation of the testicular tumour DB. Leucocytosis occurred in 81·1 per cent of these splenectomized animals (average 165,400 ; maximum 429,300 cells), as against 87·8 per cent of 49 intact animals with the same testicular tumour (average 165,100 ; maximum 517,500 cells). The splenectomy therefore did not reduce the degree of leucocytosis. In the splenectomized leucocytotic mice marked to very-marked granulopoiesis was found in 100 per cent of the lymph nodes ; in the intact animals this was 78·4 per cent. The difference is significant ($\chi^2 = 4{\cdot}42$). The other organs showed no difference in the degree of granulopoiesis between the splenectomized and the intact groups. The granulocyte production of the spleen, therefore, would seem to be taken over mainly by the lymph nodes after splenectomy.

*Forms of Granulocyte Nuclei in the Haemopoietic Organs*

In mice granulocytes arise from myeloblasts, developing via the myelocyte form to metamyelocytes, the nucleus of which initially consists in a thick smooth ring with a small lumen. On maturation of the cell this ring becomes thinner and serrated, the lumen becoming wider meanwhile. In mature cells the ring may remain closed or open up, showing lobulated structures. Petri (1933) found a predominance of closed rings in the peripheral blood of mice ; Barnes and Sisman, (1939) and De Bruyn, Korteweg and Kits van Waveren (1949) found a majority of open forms. In the haemopoietic organs of the tumour mice described here the majority of the granulocytes showed a certain type of nucleus which seemed to be specific to the organ. In the liver and the adrenals the myeloid cells often showed no maturation whatever (Fig. 5). This was particularly so in animals with the interstitial testicular tumour DB. In the case of this tumour, the total mass of myeloid cells in the periportal and capillary regions of the liver may exceed that of the liver cells ; myeloblasts and myelocytes were usually found exclusively, in some cases with a small number of young metamyelocytes. Foci of mature, granulocytes were occasionally seen, but only round the large vessels. After vital staining with trypan blue it was found that such livers contained a normal number of Kupffer cells. The granulocytes of tumour mice showed all stages from myeloblasts to mature cells in the spleen. The majority of the mature granulocytes in the spleen showed a thin annular nucleus without or with inconsiderable serration, i.e. a young maturing form (Fig. 6). In the lymph nodes and in the thymus the mature granulocytes showed more often an open, serrated type of annular nucleus, as well as S-shaped and lobulated nuclei (Fig. 7). Maturation in these lymphoid organs, therefore, reached a higher degree than that in the spleen. The often enormous masses of granulocytes in the alveolar walls and vessels in the lungs, for the most part contained hypersegmented and looped nuclei (Fig.4) The immature forms were small in number and the metamyelocytes likewise showed a deviating form in that serration was often already present in the thick ring. Mitoses were found. The same typical granulocyte nuclei were found in the lungs of a mouse without a tumour, but suffering from leucocytosis resulting from an intestinal abscess. The nuclei of the mature granulocytes in the kidney, too, were hypersegmented. The hypersegmentation possibly denotes the onset of karyorrhexis of imported granulocytes. The number of loose nuclear particles, however, is but small.

The environment in which the myeloid cells develop would, therefore, seem to influence the degree of maturation of granulocytes before these enter the circulation. This perhaps affords an explanation of the different proportions under which the nuclear types have been observed in the circulation in mice.

*Erythropoiesis*

In anaemic and polycythaemic mice extramedullary erythropoiesis occurred in the haematopoetic organs (Fig. 8, 9). In the lung too, immature red blood

| | | | | |
|---|---|---|---|---|
| No. of spleens | 32 | 59 | 53 | 49 |
| No. of l. nodes | 29 | 26 | 19 | 23 |

FIG. 11.—Erythropoiesis as related to degree of anaemia.

cells were found, but seldome more than 4 or 5 in the same alveolar wall. Fig. 11 shows the parallelism between the degree of erythropoiesis in the spleen and the lymph nodes and the degree of anaemia. There was sometimes erythropoiesis in the organs of animals without anaemia but this may well be explained by compensation of the anaemia in these animals by increased erythrocyte production. In 34 polycythaemic mice—all with the testicular tumour DB, with an average of 16·0 million R.B.C. per mm.$^3$ in normal blood volume (maximum count 34·1)—the spleen, lymph nodes, liver, lungs and adrenals were tested for erythropoiesis. In 13 of these animals there was no increased erythropoiesis in the organs in spite of the high number of R.B.C. in the peripheral blood (up to 25·5 million). Of the remaining 21 polycythaemic animals, 9 had a fairly marked erythropoiesis which, as a rule, was confined to a single organ. This moderate production cannot explain the marked increase in erythrocytes. It is known that in mice the bone marrow has very little reserve, but that some segments in the tail can be utilized

(Dunn, 1954). These sites were not investigated, so no explanation can be given for the cause of the polycythaemia.

The spleen is regarded as one of the chief breakdown sites for erythrocytes. Splenectomy would consequently be able to promote the polycythaemia. In the group of splenectomized mice, however, the frequency of polycythaemia was as high as in the group of intact mice ; the average degree of polycythaemia, too, showed no difference between the two groups. A number of splenectomized mice had anaemia ; as has been pointed out, splenectomy did not influence the abnormal blood volume either. The spleen would therefore not seem to have an unusual position among the organs involved in the haematological anomalies discussed, although it participates so actively in haemopoiesis that splenomegaly may result.

*Splenomegaly*

The spleen in tumour mice is known to be moderately enlarged as a rule. The causes of the splenomegaly in general may be oedema or hyperaemia, amyloidosis or an increase in cells. The weight of normal mouse spleens as a rule varies from 80 to 140 mg., but may be more. The average splenic weight in 472 tumour mice was 271 mg. The enlargment would seem to result mainly from increased haemopoiesis. The average weight of 20 spleens with a considerable production of both erythrocytes and granulocytes was 466 mg. Marked erythropoiesis with normal granulocyte production was found in 76 spleens with an average weight of 320 mg., whereas that of 35 spleens with a marked granulopoiesis and normal erythrocyte production was 224 mg. Thirty-eight spleens did not show abnormal haemopoiesis ; the average weight was 164 mg. i.e. hardly more than normal. Increased blood volume did not influence the splenic weight. Splenomegaly due to cavitary dilatations (see later) was rare. The part of an increase in the megakaryocyte count in splenomegaly was not determined; this was impossible because an abnormally high megakaryocyte count was nearly always associated with haemopoietic activity.

*Megakaryocytes*

The megakaryocytes in the spleen showed a marked increase in 224 of 472 tumour mice. Megakaryocytes were also often found in the liver (up to 70 in a single section of 9 sq. mm.), and exceptionally also in the lungs, lymph nodes and adrenals. There is a difference of opinion as regards the origin of the megakaryocytes in the spleen ; they are believed to develop from a basophile lymphoid mother cell, (Klemperer, 1938), or directly from myeloblasts (Ham, 1950). In the latter case an increase in megakaryocytes could be expected to be associated with myelopoiesis in the spleen of tumour mice. In 81 of the 224 spleens rich in megakaryocytes, however, there was no increased granulopoiesis ; in only 52 spleens was a high megakaryocyte count associated with marked granulocyte production. Combination with marked erythropoiesis was found significantly more often (in 71 of 224 spleens), nut erythropoietic activity was absent in 63 spleens with abundant megakaryocytes. Lymphoid hyperplasia was never observed in our material. No relationship between megakaryocytes and other blood cells in the spleen was therefore demonstrable.

*Cavitary congestive dilatations*

Furth and Boon (1945) made mention of cavitary dilatations in the liver in mice bearing a transplanted granulosa-cell tumour. These congestive sinusoidal dilatations associated with granulosa-cell tumours producing increased blood volume have since also been found in the spleen, adrenals and ovaries (Furth and Sobel, 1946 ; Cliffton and Wolstenholme, 1949 ; Bali and Furth, 1949 ; Furth and Moshman, 1951). The same phenomenon was encountered in mice bearing a transplanted interstitial testicular tumour (Wolstenholme and Gardner, 1950). A correlation with increased blood volume was found. The findings of the above-mentioned authors were confirmed in that cavitary dilatations in the organs were only found in animals with increased blood volume. A correlation was found between the degree of the anomaly and the degree of increased blood volume. Slight dilatations—periportal in the liver and as a rule in the periphery of the adrenal cortex—became visible once the plasma volume had increased to about 1·50 × its original value ; marked spongy changes occurred at about 3·75 × the normal plasma volume (Fig. 10). In 17 per cent of the animals with a plasma volume increased to more than twice its original value no congestive changes were seen. However, the ovaries were not examined and the anomaly was sometimes confined to a single organ. No other correlation than that with the increased blood volume was found for the sinusoidal congestive dilatations.

---

EXPLANATION OF PLATES.

FIG. 4.—Granulocytes in lung. × 475. Mature nuclei hypersegmented and looped. Salivary gland tumour BDf (34th transplantation). Tumour weight : 7·8 g. Leucocyte count in peripheral blood computed on normal blood volume : 106,300/c.mm.

FIG. 5.—Immature granulocytes in liver. × 475. Testicular tumour DB (24th transplantation). Tumour weight : 9·3 g. Leucocyte count in peripheral blood computed on normal blood volume : 490,500/c.mm.

FIG. 6.—Granulocytopoiesis in spleen. × 475. Mature nuclei mostly closed-ring-shaped. Testicular tumour DB (28th transplantation). Tumour weight : 6·3 g. Leucocyte count in peripheral blood computed on normal blood volume : 53,600/c.mm.

FIG. 7.—Granulocytopoiesis within medullary cords of lymph node. × 475. Shapes of mature nuclei : closed-rings, open-serrated-rings and loops. Testicular tumour DB (24th transplantation). Tumour weight : 6·8 g. Leucocyte count in peripheral blood computed on normal blood volume : 55,400/c.mm.

FIG. 8.—Erythropoiesis in liver. × 250. Nucleated red cells within the capillaries. Adrenal cortical carcinoma DO (14th transplantation). Tumour weight : 5·6 g.
Blood counts computed on normal blood volume

| | |
|---|---|
| Haematocrit | 0·68 × normal value. |
| R.B.C./c.mm. | 0·35 × ,, ,, |
| Cell volume $\mu^3$ | 1·93 × ,, ,, |
| Haemoglobin | 0·56 × ,, ,, |
| Hb concentration | 0·79 × ,, ,, |

FIG. 9.—Erythropoiesis in kidney. × 250. Nucleated red cells within the glomerulus. Testicular tumour Af (13th transplantation). Tumour weight : 4·2 g.
Blood counts computed on normal volume :

| | |
|---|---|
| Haematocrit | 2·06 × normal value. |
| R.B.C./c.mm. | 1·69 × ,, ,, |
| Cell volume $\mu^3$ | 1·27 × ,, ,, |
| Haemoglobin | 1·89 × ,, ,, |
| Hb concentration | 0·92 × ,, ,, |

FIG. 10.—Sinusoidal dilatation in liver. × 250. Immature granulocytes and nucleated red cells within sinusoids. Testicular tumour DB (19th transplantation). Plasma volume : 4·74 × normal value. Tumour weight : 7·5 g.

4

5

6

7

Tengbergen and Mühlbock.

8

9

10

Tengbergen and Mühlbock.

SUMMARY

An investigation was made into the effect of spontaneous and transplanted tumours on the blood in mice of various strains. The material investigated included 1264 tumour mice, of which 31 had spontaneous mammary tumours, while 1233 were bearing transplanted tumours of varying origin. These tumours, to a total of 76, included 44 mammary tumours, 2 interstitial testicular tumours, 5 adrenocortical tumours, 5 hepatomata, 2 squamous gastric tumours, 3 maxillary sarcomatoid carcinomata, 1 mixed tumour of the salivary glands, 3 pulmonary tumours, 4 uterine sarcomata, 3 fibro-sarcomata, 2 myxosarcomata and 2 haemangiomata.

The interpretation of haematological values in the tumour mice was based on determinations of the normal blood volume in 1840 untreated mice of the same strains as the tumour mice. The normal blood volume proved to be dependent on the strain, the body weight and sometimes on the sex of the animals.

Many tumour mice proved to have increased blood volumes, as a rule plasmatic but sometimes also polycythaemic or exclusively so. After correction of the cell and Hb values found (by conversion based on the normal blood volume), the tumour mice were found to have one or more of the following disorders: haemolytic anaemia, symptomatic polycythaemia and leukaemoid reaction.

Splenectomy had no inhibitory or stimulating effect on any of the haematological anomalies.

Causative factors were studied; necrosis of the tumour was found to be of little or no importance in this respect; nor was the size of the tumour significant. Sex hormones produced by the tumours probably constituted no causative, but may be a promoting, factor for increased blood volume and polycythaemia.

Morphological examination of the haemopoietic organs revealed granulopoiesis in accordance with the degree of leucocytosis of the peripheral blood. Erythropoiesis corresponded to the degree of anaemia but afforded no explanation of the polycythaemia.

An increase in the megakaryocyte count of the spleen in tumour mice was not associated with a parallel marked degree of granulopoiesis or erythropoiesis.

In the granulopoietic organs a predominance was found of a myeloid-cellular nuclear type typical of each organ.

The usually moderate splenomegaly in tumour mice was attributable chiefly to increased haemopoiesis.

Congestive sinusoidal dilatations in the liver and the adrenals were only found in animals showing increased blood volume.

### REFERENCES

BALI, T. AND FURTH, J.—(1949) *Cancer Res.*, **9**, 449.

BARNES, W. A. AND SISMAN, J. E.—(1939) *Amer. J. Cancer*, **37**, 1.

BATEMAN, J. C.—(1951*a*) *J. nat. Cancer Inst.*, **11**, 671.—(1951*b*) *Blood*, **6**, 639.

BRUYN, W. M. DE, KORTEWEG, R. AND WAVEREN, E. KITS, VAN.—(1949) *Cancer Res.*, **9**, 282.

BURKE, J. D.—(1954) *Phys. Zool.*, **27**, 1.

CLIFFTON, E. E. AND WOLSTENHOLME, M. D.—(1949) *Cancer Res.*, **9**, 331.

CONTOPOULUS, A. N., ELLIS, S., SIMPSON, M. E., LAWRENCE, J. H. AND EVANS, H. M. —(1954) *Endocrinology*, **55**, 808.

COURTICE, F. C.—(1943) *J. Physiol.*, **102**, 290.
DUNN, TH. B.—(1954) *J. nat. Cancer Inst.*, **14**, 1281.
DYKE, D. C. VAN, CONTOPOULUS, A. N., WILLIAMS, B. S., SIMPSON, M. E. AND LAWRENCE, J. H.—(1954) *Acta haemat.*, **11**, 203.
FURTH, J.—(1946) *Proc. Soc. exp. Biol. N.Y.*, **61**, 212.
*Idem*, BEALE, E. J., WISH, L. AND KNOOHUIZEN, M. M.—(1951) *Cancer Res.*, **11**, 249.
*Idem* AND BOON, M. C.—(1945) *Proc. Soc. exp. Biol. N.Y.*, **58**, 112.
*Idem* AND MOSHMAN, J.—(1951) *Cancer Res.*, **11**, 543.
*Idem* AND SOBEL, H.—(1946) *J. nat. Cancer Inst.*, **7**, 103.
GOTTSCHALK, R. AND FURTH, J.—(1951) *Acta haemat.*, **5**, 100.
GUSSIO, S.—(1911–12) *Tumori*, **1**, 661.
HAM, A. W.—(1950) ' Histology '. Philadelphia (Lippincott).
HENSLER, L.—(1953) *Schweiz. med. Wschr.*, **83**, 1032.
KLEMPERER, P.—(1938) ' Handbook of Hematology '. New York (Hoeber), p. 1591.
MÜHLBOCK, O.—(1952) *Geburtsh. u. Frauenheilk.*, **12**, 289.
NIE, R. VAN.—(1957) Thesis, Utrecht.
PAPPENHEIM, A.—(1911) *Folia haemat.*, **10**, 393.
PETRI, S.—(1933) *Acta path. microbiol. scand.*, **10**, 159.
PONDER, E. AND NESMITH, J.—(1952) *Cancer Res.*, **12**, 104.
QUERIDO, A. AND OVERBEEK, A.—(1938) *Arch. int. Pharmacodyn.*, **59**, 370.
RUSSELL, E. S., NEUFELD, E. F. AND HIGGINS, C. T.—(1951) *Proc. Soc. exp. Biol. N.Y.*, **78**, 761.
SOBEL, H. AND FURTH, J.—(1948) *Endocrinology*, **42**, 436.
WEISBERGER, A. S., QUYTON, R. A., HEINLE, R. W. AND STORAASLI, J. P.—(1951) *Blood*, **6**, 916.
WISH, L., FURTH, J. AND STOREY, R. H.—(1950) *Proc. Soc. exp. Biol. N.Y.*, **74**, 644.
WOLSTENHOLME, J. T. AND GARDNER, W. U.—(1950) *Ibid.*, **74**, 659.